# Supplementary material for: Dual-plasmonic Au@Cu7S4 yolk@shell nanocrystals for photocatalytic hydrogen production across visible to near infrared spectral region
Source: Nat Commun. 2024 Jan 9;15:413. doi: 10.1038/s41467-023-44664-3 (PMC10776726; doi:10.1038/s41467-023-44664-3)
Supplement: Supplementary file 1 — Supplementary Information [file 41467_2023_44664_MOESM1_ESM.pdf]

Supplementary information

Dual-plasmonic Au@Cu_7S_4 yolk@shell nanocrystals for photocatalytic hydrogen production across visible to near infrared spectral region

Chun-Wen Tsao,[1] Sudhakar Narra,[2] Jui-Cheng Kao,[1] Yu-Chang Lin,[3] Chun-Yi Chen,[4] Yu-Cheng Chin,[5] Ze-Jiung Huang,[5] Wei-Hong Huang,[6] Chih-Chia Huang,[5] Chih-Wei Luo,[3,6,7] Jyh-Pin Chou,[8] Shigenobu Ogata,[9] Masato Sone,[4] Michael H. Huang,[10] Tso-Fu Mark Chang,[4,*] Yu-Chieh Lo,[1,*] Yan-Gu Lin,[3,*] Eric Wei-Guang Diau,[2,11,*] Yung-Jung Hsu[1,11,12,*]

[1] Department of Materials Science and Engineering, National Yang Ming Chiao Tung University, Hsinchu 300093, Taiwan
[2] Department of Applied Chemistry and Institute of Molecular Science, National Yang Ming Chiao Tung University, Hsinchu 300093, Taiwan
[3] National Synchrotron Radiation Research Center, Hsinchu 30076, Taiwan
[4] Institute of Innovative Research, Tokyo Institute of Technology, Kanagawa 226-8503, Japan
[5] Department of Photonics, National Cheng Kung University, Tainan 70101, Taiwan
[6] Department of Electrophysics, National Yang Ming Chiao Tung University, Hsinchu 300093, Taiwan
[7] Institute of Physics, National Yang Ming Chiao Tung University, Hsinchu 300093, Taiwan
[8] Department of Physics, National Changhua University of Education, Changhua 50007, Taiwan
[9] Department of Mechanical Science and Bioengineering, Osaka University, Toyonaka 560-8531, Japan
[10] Department of Chemistry, National Tsing Hua University, Hsinchu 30013, Taiwan
[11] Center for Emergent Functional Matter Science, National Yang Ming Chiao Tung University, Hsinchu 300093, Taiwan
[12] International Research Frontiers Initiative, Institute of Innovative Research, Tokyo Institute of Technology, Kanagawa 226-8503, Japan

*E-mail: chang.m.aa@m.titech.ac.jp (T.-F. M. Chang); yclo@nycu.edu.tw (Y.-C. Lo); lin.yg@nsrrc.org.tw (Y.-G. Lin); diau@nycu.edu.tw (E. W.-G. Diau); yhsu@nycu.edu.tw, yhsu@cc.nctu.edu.tw (Y.-J. Hsu)

Content list

1. Supplementary results and discussion

2. Supplementary figures

3. Supplementary tables

4. Supplementary references

1. Supplementary results and discussion

Optical properties and band structure

The optical properties of the samples were studied with UV-visible-NIR absorption spectroscopy. As shown in Supplementary Fig. 1b, the three Au@Cu_7S_4 and pure Cu_7S_4 exhibited similar absorption characteristics, with one prominent absorption edge extending to the visible region and the other broad absorption band spanning the NIR region. The visible absorption edge at approximately 600 nm was attributed to the bandgap absorption of the Cu_7S_4 component, which corresponded to an optical bandgap of 2.05 eV, as estimated from the corresponding Tauc plot (Supplementary Fig. 22a). The absorption onset at 750 nm was ascribed to the sub-gap absorption associated with the defect levels induced by Cu vacancies.[1,2] The broad band ranging from 750 nm to wavelengths beyond 2200 nm, on the other hand, originated from the LSPR absorption of Cu_7S_4 as a result of Cu deficiency. It should be noted that Au@Cu_7S_4 did not exhibit an obvious spectral feature that can be identified as the LSPR band of Au. For pure Au colloids, the LSPR was located at 520 nm (Supplementary Fig. 22b). A redshift in the LSPR band of Au was expected for Au@Cu_7S_4 because of the larger refractive index of the surrounding Cu_7S_4 ($n = 1.4$ to 3.3)[3] than that of the solvent ($n = 1.33$ for water). The disappearance of an identifiable LSPR band of Au for Au@Cu_7S_4 might derive from the spectral overlap with the strong absorption tail of Cu_7S_4. Although the LSPR band of the Au component for Au@Cu_7S_4 was not experimentally identified, it can be theoretically examined with the empirical equation derived from Mie theory.

The LSPR absorption of noble metal nanostructures is strongly influenced by factors associated with the surrounding environments, such as the thickness of the neighboring dielectric and the refractive index of

the solvent medium.[4] An empirical equation derived from Mie theory has been proposed to compute the theoretical LSPR position of Au in the dielectric environment.[5,6] In this equation, the volume fractions of the constituent components are decisive terms dictating the LSPR position of Au. For the current Au@Cu_7S_4, the volume fraction of Au (f_{Au}) was considerably lower than unity (f_{Au} = 0.032 for 5-Au@Cu_7S_4); in other words, the shell surrounding Au yolk was so thick that the LSPR absorption of Au was solely mediated by the thick shell, and the solvent medium only posed a fairly minor impact on the overall LSPR absorption. With this consideration, the empirical equation for computing the LSPR position of Au (λ_{LSPR}) can be expressed as Equaiton (1):[6]

$$\lambda_{LSPR}^2 = \lambda_p^2 \left\{ \varepsilon_\infty + 2n_{shell}^2 \left[\frac{(n_{shell}^2 + 2n_{solvent}^2) + f_{Au} \times (n_{solvent}^2 - n_{shell}^2)}{n_{shell}^2 + 2n_{solvent}^2} \right] \right\} \qquad (1)$$

Here, λ_p is the bulk plasma wavelength of Au, which is 130.9 nm,[7] ε_∞ is the high-frequency dielectric constant of Au, which is approximately 12,[8] f_{Au} is the volume fraction of Au, and n_{shell} and $n_{solvent}$ represent the refractive index of the shell component (n_{shell} = 1.4 to 3.3 for Cu_7S_4)[3] and solvent medium ($n_{solvent}$ = 1.33 for water), respectively. The computed LSPR wavelength of 5-Au@Cu_7S_4 was located in the range from 522.2 to 755.8 nm corresponding to the divergent refractive index of Cu_7S_4 from 1.4 to 3.3. Since the Au yolk particles were movable inside the Cu_7S_4 hollow shell, the interaction of Au with the surrounding Cu_7S_4 and its influence on the LSPR shift could be compromised.[9-11] The polycrystalline, defective nature of the Cu_7S_4 shell could also reduce the extent of the LSPR shift owing to the decrease in the effective refractive index of the surrounding environment.[12] As a result, the shift of the experimental LSPR might become less

substantial than expected. In other words, the experimental LSPR of Au@Cu_7S_4 could be located at wavelengths shorter than expected, making the identification of the LSPR band and its differentiation from the absorption tail of Cu_7S_4 improbable.

Supplementary Fig. 1c compares the steady-state PL spectra among the three Au@Cu_7S_4 and pure Cu_7S_4. These samples all exhibited a prominent PL emission centered at approximately 525 nm, consistent with the reported PL features of $Cu_{2-x}S$ quantum dots.[13] Compared with pure Cu_7S_4, the three Au@Cu_7S_4 showed a depressed PL intensity. This outcome suggested that pronounced charge separation occurred at the interface between Au and Cu_7S_4, prohibiting electron-hole recombination to depress the PL intensity of the Cu_7S_4 component. To better interpret how charge separation occurred, UPS measurements were carried out on the constituted components, *i.e.*, pure Au and pure Cu_7S_4, with the aim of depicting the possible band alignment for Au@Cu_7S_4. Supplementary Figs. 22c and 22d show the UPS spectra recorded for pure Cu_7S_4 in the high-energy region and in the region close to 0 eV, respectively. The spectrum in the high-energy region revealed a cut-off energy of 17.05 eV. By subtracting the cut-off energy from the incident photon energy (21.22 eV), the apparent work function of pure Cu_7S_4 can be computed as 4.17 eV. The spectrum in the region close to 0 eV, on the other hand, contains information on the valence band level (E_{VB}) of the sample relative to the Fermi level (E_F). By applying a linear extrapolation to the tangent of the onset, the distance of E_{VB} from the E_F can be received, approximately 0.69 eV for pure Cu_7S_4. By adding the apparent work function value, the E_{VB} of pure Cu_7S_4 with respect to vacuum was determined to be −4.86 eV. Further addition of the optical bandgap can give the conduction band level (E_{CB}) with respect to vacuum, which was estimated to be −2.81 eV for pure Cu_7S_4. By using the same calculation procedure, the apparent work

function of pure Au can also be obtained from Supplementary Fig. 22e, giving an E_F value of −4.29 eV with respect to vacuum. With these energy levels, a plausible band alignment for Au@Cu_7S_4 to interpret the interfacial charge transfer pathways is depicted in Supplementary Fig. 1d. For Au@Cu_7S_4, as Cu_7S_4 and Au were brought in contact, the lower E_F of Au induced an upward bending of the band edge in Cu_7S_4 as a result of the depletion of electrons. Upon band edge excitation, the upward band bending at the interface facilitated photoexcited hole transfer from Cu_7S_4 to Au and enabled the photogenerated electrons to be concentrated in Cu_7S_4. Because the photoexcited holes were separated from the photogenerated electrons, radiative electron-hole recombination could be reduced to cause a depressed PL intensity for Au@Cu_7S_4.

Chemical and structural stability

As revealed in Supplementary Fig. 5, no appreciable change in microstructural features or chemical state could be observed for 5-Au@Cu_7S_4 upon the extended hydrogen production operation. Importantly, no noticeable Cu^{2+} satellite peaks could be observed after 30 hours of hydrogen production, signifying that the Cu_7S_4 component was not corrosively oxidized during the photocatalytic reaction. To further corroborate the exclusive existence of Cu^{1+}, Auger electron spectroscopic analysis was carried out on 5-Au@Cu_7S_4 before and after photocatalysis. As shown in Supplementary Fig. 5e, the Cu *LMM* spectrum for 5-Au@Cu_7S_4 before and after photocatalysis was nearly identical, exhibiting a peak centered around 916.4 to 916.6 eV. This kinetic energy can be convincingly assigned to Cu^{1+}.[14] In order to confirm the high chemical and structural stability, HRTEM and EDS mapping analysis were further conducted on 5-Au@Cu_7S_4 after used in hydrogen production for 30 successive hours. As showed in Supplementary Fig. 6, the crystallographic

structure and elemental composition of the used 5-Au@Cu_7S_4 remained unchanged. Besides, the void size (27.0 ± 2.6 nm), shell thickness (11.6 ± 0.9 nm) and Au size (15.1 ± 1.0 nm) of the used sample were also examined, showing nearly identical size distribution to that of the as-prepared sample (void size = 26.5 ± 3.0 nm; shell thickness = 11.7 ± 1.5; Au size = 15.2 ± 0.6 nm). This outcome corroborated the high chemical and structural stability for Au@Cu_7S_4 toward solar hydrogen production.

Thermal effect and photothermal property

The thermal effect induced by light irradiation has also been investigated by recording the temperature profiles of the electrolyte containing 5-Au@Cu_7S_4 before and after for 30 successive hours of AM 1.5 G illumination. As Supplementary Fig. 7 reveals, upon 30 successive hours of light irradiation, the temperature of the 5-Au@Cu_7S_4-contained electrolyte increased from 27.1 to 28.4 ^{o}C, whereas the temperature of pure electrolyte increased from 27.2 to 27.6 ^{o}C during the same irradiation period. Such a minimal temperature rise was far less than the temperature required for proceeding with photochemical hydrogen production, even when a sophisticated catalyst is present (higher than 100 ^{o}C).[15,16] Previous study also suggested that the thermal energy induced by light irradiation could barely promote photocatalytic reactions in a liquid-solid heterogeneous system due to the rapid heat dissipation to the surrounding liquid.[17] To validate this contention, additional photocatalytic experiments on 5-Au@Cu_7S_4 by controlling the electrolyte temperature were performed. As displayed in Supplementary Fig. 8, by introducing a cold plate, the electrolyte temperature can maintain a nearly constant value throughout the photocatalytic reaction process. The resultant hydrogen production performance was compared with that obtained without temperature control. The difference in hydrogen production rate between the two conditions (under temperature control and

without temperature control) was 3.6 % based on the results of four duplicate sets of experiments. This outcome validated that the thermal effect on hydrogen production can be considered rather minor in the current photocatalytic system. This suggestion, however, did not imply the neglect of photothermal effect itself. In fact, the current Au@Cu_7S_4 is anticipated to exhibit photothermal effect since both Au and Cu_7S_4 are capable of depositing thermal energy to lattice vibrations upon LSPR excitation. Nevertheless, the observed temperature rise for 5-Au@Cu_7S_4-contained electrolyte was fairly limited. The cause was believed to be associated with the considerably large volume of the electrolyte (40.0 mL) and relatively low power of light irradiation (100 mW cm^{-2}) employed in photocatalytic reactions. Note that photothermal effect in terms of a substantial rise in solution temperature can only be experimentally probed when the sample is dispersed in a solvent of small volume (from hundreds of μL to a few mL) and irradiated with a laser of high power (from a few W cm^{-2} to tens of W cm^{-2}).[18-23] In a previous study, an noticeable temperature increase to around 40 ^{o}C was observed for a 2.0 mL aqueous solution containing Au@$Cu_{1.5}S$ core@shell nanoparticles under laser irradiation (λ = 980 nm, power = 6.25 W cm^{-2}).[19] In the other study, a pronounced temperature elevation to 52.2 ^{o}C was recorded on a 1.0 mL aqueous dispersion of Au nanorod@Cu_7S_4 nanooctahedron yolk@shell particles upon laser irradiation (λ = 915 nm, power = 2.12 W cm^{-2}).[18] In a different work, a substantial temperate rise to 72.9 ^{o}C was achieved by irradiating Au@$Cu_{2-x}S$ core@shell nanorod suspension with light covering the whole infrared spectrum (λ = 700-2000 nm, power = 1 W cm^{-2}).[20] These demonstrations showed that a small volume of sample solution accompanied with an intense laser irradiation is required to achieving noticeable temperature increase caused by photothermal heating. This requirement is particularly indispensable to plamonic materials because plamonic heating is localized at the sample surface

and is only effective in the vicinity of sample surface. If a plamsonic material is dispersed in a large medium and irradiated by a weak light, the generated thermal energy is limited and cannot effectively heat up the whole surrounding medium. This explained why a minimal temperature rise was observed for 5-Au@Cu_7S_4-contained electrolyte in the current photocatalytic system, in which a large electrolyte volume and a low irradiation power were set.

To examine the data credibility of thermal effect, we have recorded the temperature profiles of the electrolytes containing pure Cu_7S_4 or pure Au under AM 1.5 G illumination without temperature control. Supplementary Figs. 10-13 summarize the temperature profiles for all of the electrolytes taken at a given time interval of irradiation. The electrolytes containing pure Au or pure Cu_7S_4 also exhibited a fairly limited temperature increase (less than 1.5 ^{o}C) upon 30 successive hours of light irradiation. This outcome was expectable because of the employment of a large volume of electrolyte and a low power of irradiation. The consistency of the recorded temperature profiles for all of the electrolytes further ensured the reproducibility of the experiments. In order to highlight the relevance of irradiation power, we have also recorded the temperature profiles of 5-Au@Cu_7S_4-contained electrolyte under two-sun (200 mW cm^{-2}) and three-sun (300 mW cm^{-2}) irradiation conditions As Supplementary Fig. 14 compares, the temperature rise of 5-Au@Cu_7S_4-contained electrolyte upon 6 hours of one-sun, two-sun and three-sun irradiation respectively reached 1.5, 2.8 and 4.5 ^{o}C. The accordingly increased extent of temperature rise with increasing irradiation power revealed the importance of irradiation power in heating up the whole electrolyte surrounding Au@Cu_7S_4. This finding also stood up for the argument that a small volume of sample solution accompanied with an intense laser irradiation is required to achieving noticeable temperature increase

caused by photothermal heating.

For the current Au@Cu_7S_4, the temperature rise of the electrolyte induced by photothermal heating can reach a level approximating to those of Au and $Cu_{2-x}S$-based heterostructures reported in the literature (from 40 to 72.9 °C),[18-20] provided that the required experimental conditions, i.e. electrolyte volume and irradiation power, are applied. To examine the intrinsic features of photothermal heating, photothermal experiments by irradiating 0.2 mL of 5-Au@Cu_7S_4-contained electrolyte with high-power lasers were conducted under various excitation wavelengths from visible to NIR region. Supplementary Fig. 15 displays the resultant temperature evolutions under various irradiation conditions. Both visible (λ = 532 nm and 650 nm) and NIR excitations (λ = 785 nm, 808 nm, and 1064 nm) caused a perceivable, gradual temperature increase with irradiation time. The temperature rising processes can be visualized from the corresponding thermograph images showed in Supplementary Fig. 16. Notably, under 808 nm laser excitation (power = 2.0 W cm^{-2}) for 20 min, the electrolyte temperature of 5-Au@Cu_7S_4 can exceed 57 °C as a result of the prevalence of pronounced photothermal effect. This observation further corroborated the argument that a small volume of sample solution accompanied with an intense laser irradiation is required to achieving noticeable temperature increase caused by photothermal heating. It is important to note that the achievable temperature rise for photothermal materials is highly sensitive to the experimental conditions,[19,21,22,24] such as the concentration of the dispersed photothermal materials, the wavelength and power of the irradiation, as well as the volume of the solution. As an illustration, the photothermal performance of 5-Au@Cu_7S_4-contained electrolyte (0.2 mL) has also been examined under 808 nm irradiation with a power set to be equal to the irradiance of the standard AM 1.5 G spectra (0.11 mW cm^{-2} at 808 nm as determined from ASTM G-173093

data set). As showed in Supplementary Fig. 17, the electrolyte temperature nearly unchanged despite the use of a small electrolyte volume. Additional comparative experiment was further performed by irradiating 5-Au@Cu_7S_4-contained electrolyte (0.2 mL) at an irradiation wavelength where standard AM 1.5 G spectra show peak irradiances (around 0.15 mW cm^{-2} at 440 nm). The electrolyte temperature did not vary as well. These illustrations reflected that the visible and NIR photons of AM 1.5 G illumination were essentially ineffective for causing a noticeable temperature rise for 5-Au@Cu_7S_4 induced by photothermal heating even though a small volume of electrolyte was employed. This observation also supported our explanations on the cause for the minimal temperature rise observed for 5-Au@Cu_7S_4-contained electrolyte in the current photocatalytic system, in which a large electrolyte volume and a low irradiation power were set. The dependence of photothermal performance on experimental conditions has made the direct comparison of the temperature rise of the current Au@Cu_7S_4 with those reported in other $Cu_{2-x}S$-based photothermal systems improbable. We believe there is definitely room for the further improvement of photothemal performance for the current Au@Cu_7S_4 by optimizing the experimental factors.

Previous development of dual-plamsonic heterostructures

In recent years, dual-plasmonic heterostructures comprising plasmonic metals and plasmonic semiconductors have been widely investigated due to the intriguing optical properties resulting from the synergy of the two LSPR features.[25,26] Previous studies have demonstrated the extensive use of dual-plasmonic heterostructures in photothermal and biomedical applications. For photocatalytic applications, using dual-plasmonic heterostructures as photocatalysts is still in its infancy. Supplementary Table 7 summarizes the recent development of dual-plasmonic photocatalysts and the scenarios of their

photocatalytic applications. Most of the reaction scenarios ever demonstrated on dual-plasmonic photocatalysts were the degradation of organic dyes. For NIR-driven hydrogen production, only one practice has been made on Au/CuSe tangential hybrids, showing 0.34 % of AQY of hydrogen production at 940 nm irradiation in the presence of Pt co-catalyst.[27] As a comparison, the current Au@Cu_7S_4 has achieved an advanced AQY of 2.7 % at 900 nm and a record-breaking AQY of 7.3 % at 2200 nm in the absence of additional co-catalysts. This achievement has never been realized by previously reported dual-plasmonic photocatalysts and has surpassed the performance of other state-of-the-art NIR-responsive photocatalysts.

2. Supplementary figures

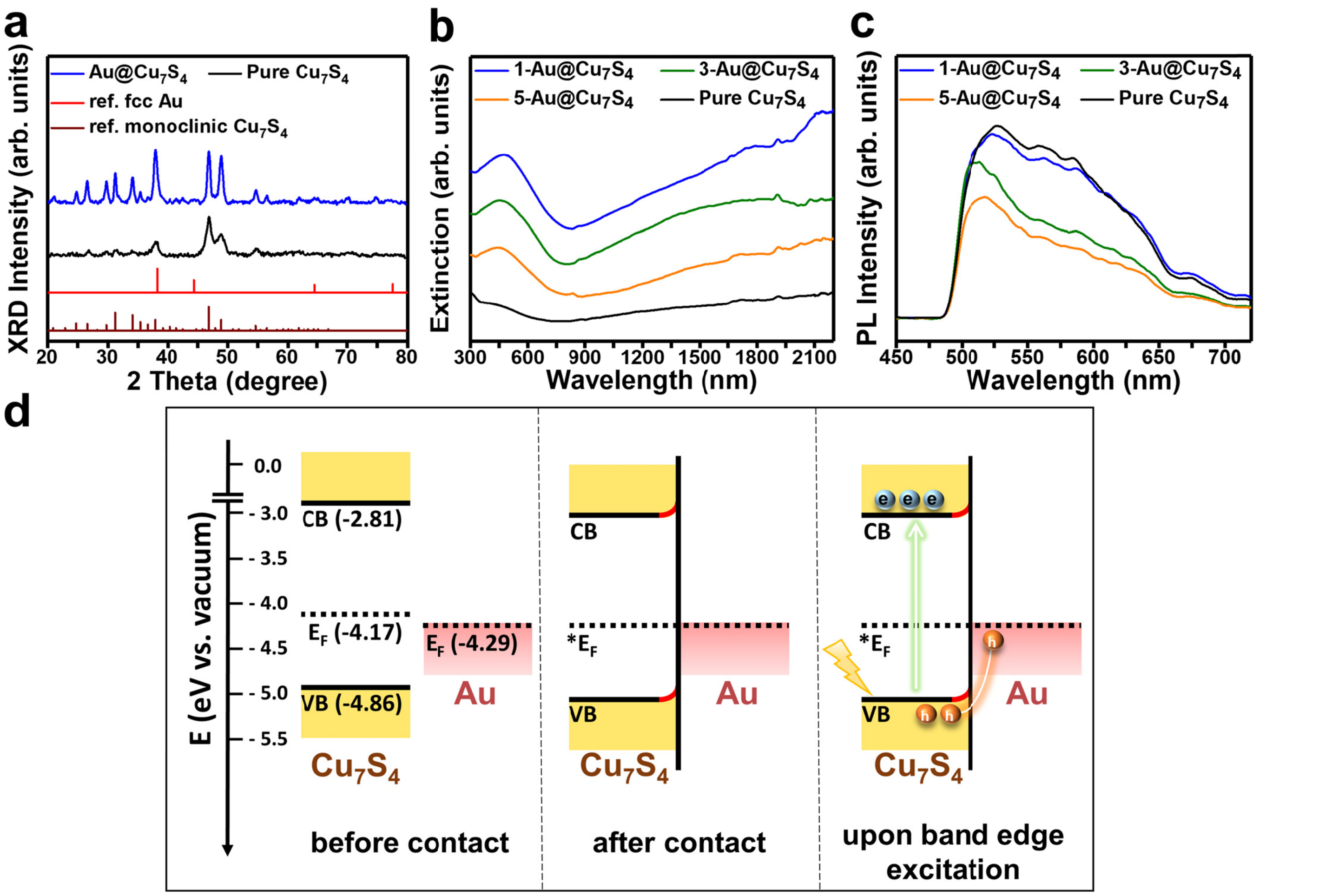

Supplementary Fig. 1 Crystallographic structure and optical properties of Au@Cu_7S_4. **a** XRD patterns, **b** UV-visible-NIR absorption spectra, **c** Steady-state PL spectra of pure Cu_7S_4 and the three Au@Cu_7S_4. In **a**, the standard patterns of fcc Au (PDF #04-0784) and monoclinic Cu_7S_4 (PDF #23-0958) were also included. **d** Proposed band alignment and interfacial charge transfer scenario for Au@Cu_7S_4 under band edge excitation.

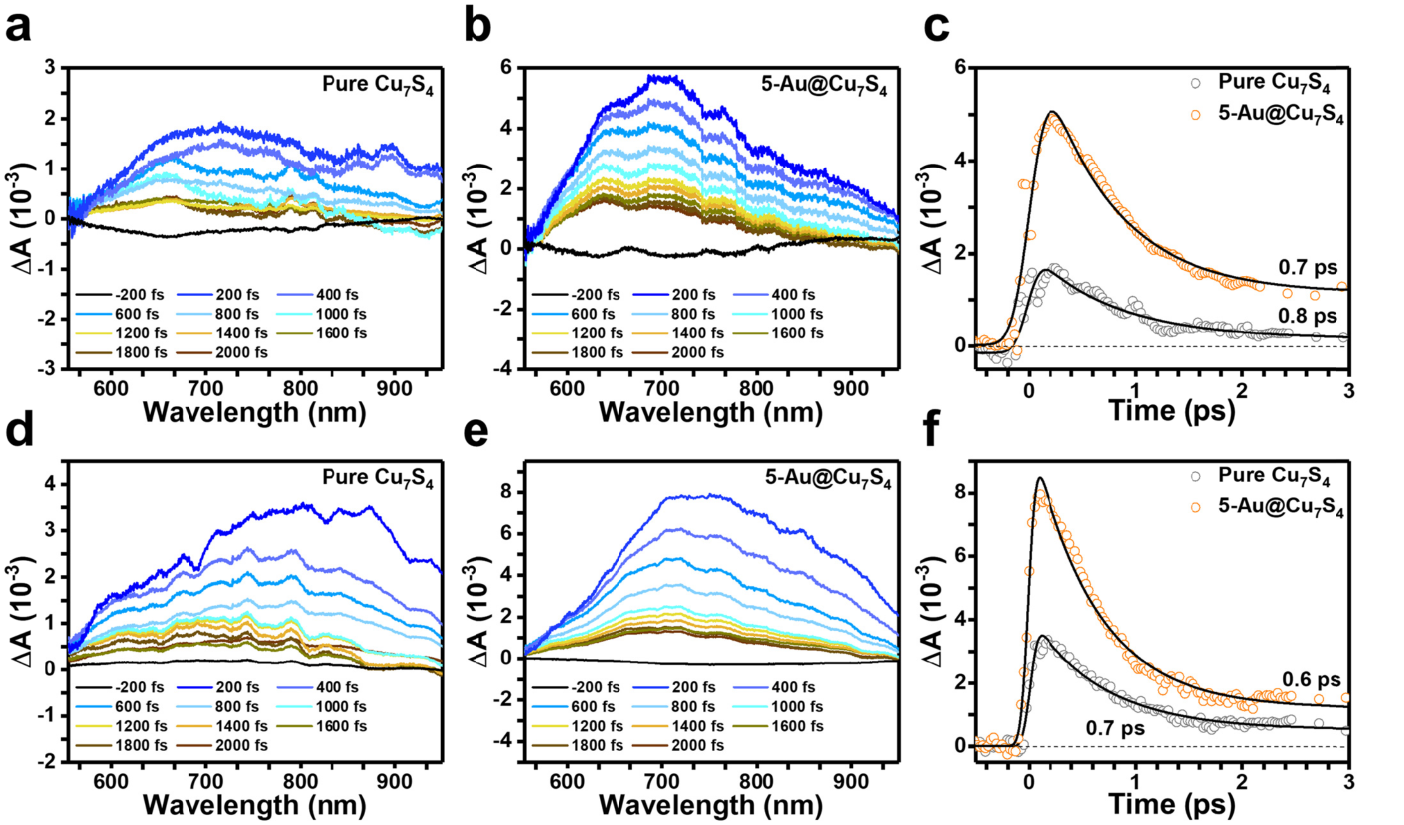

Supplementary Fig. 2 Femtosecond TAS. Spectral and temporal profiles for pure Cu_7S_4 and 5-Au@Cu_7S_4 measured by using excitation wavelength of **a-b** 500 nm and **d-e** 1400 nm. The laser fluences for 500 and 1400 nm excitations were set to 5 and 0.7 mJ/cm^2 respectively. Knietic pofiles for pure Cu_7S_4 and 5-Au@Cu_7S_4 measured by using excitation wavelength of **c** 500nm and **f** 1400 nm. **c** was obtained by probing at 660 nm for both pure Cu_7S_4 and 5-Au@Cu_7S_4. **f** was obtained by probing at 740 nm for pure Cu_7S_4 and 700 nm for 5-Au@Cu_7S_4.

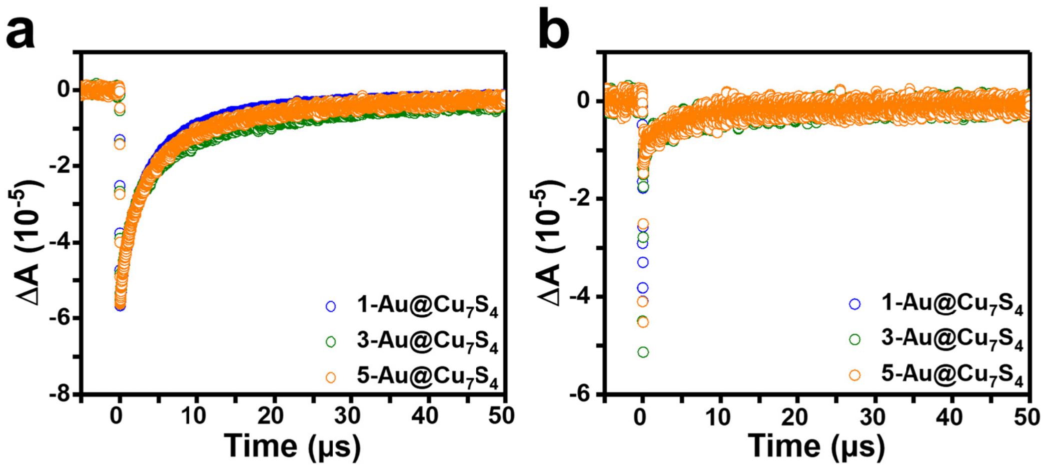

Supplementary Fig. 3 Nanosecond TAS. Kinetics profiles for the three Au@Cu_7S_4 measured by using excitation wavelength of **a** 532 nm and **b** 1064 nm and integrating the area under the intensity curves from 900 to 2400 nm.

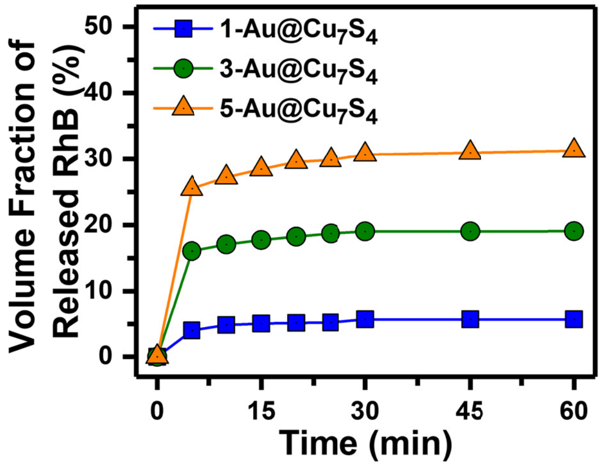

Supplementary Fig. 4 RhB release experiments. RhB was used as optical probe to estimate the mass transport kinetics of reacting/product species across the shell for the three Au@Cu_7S_4.

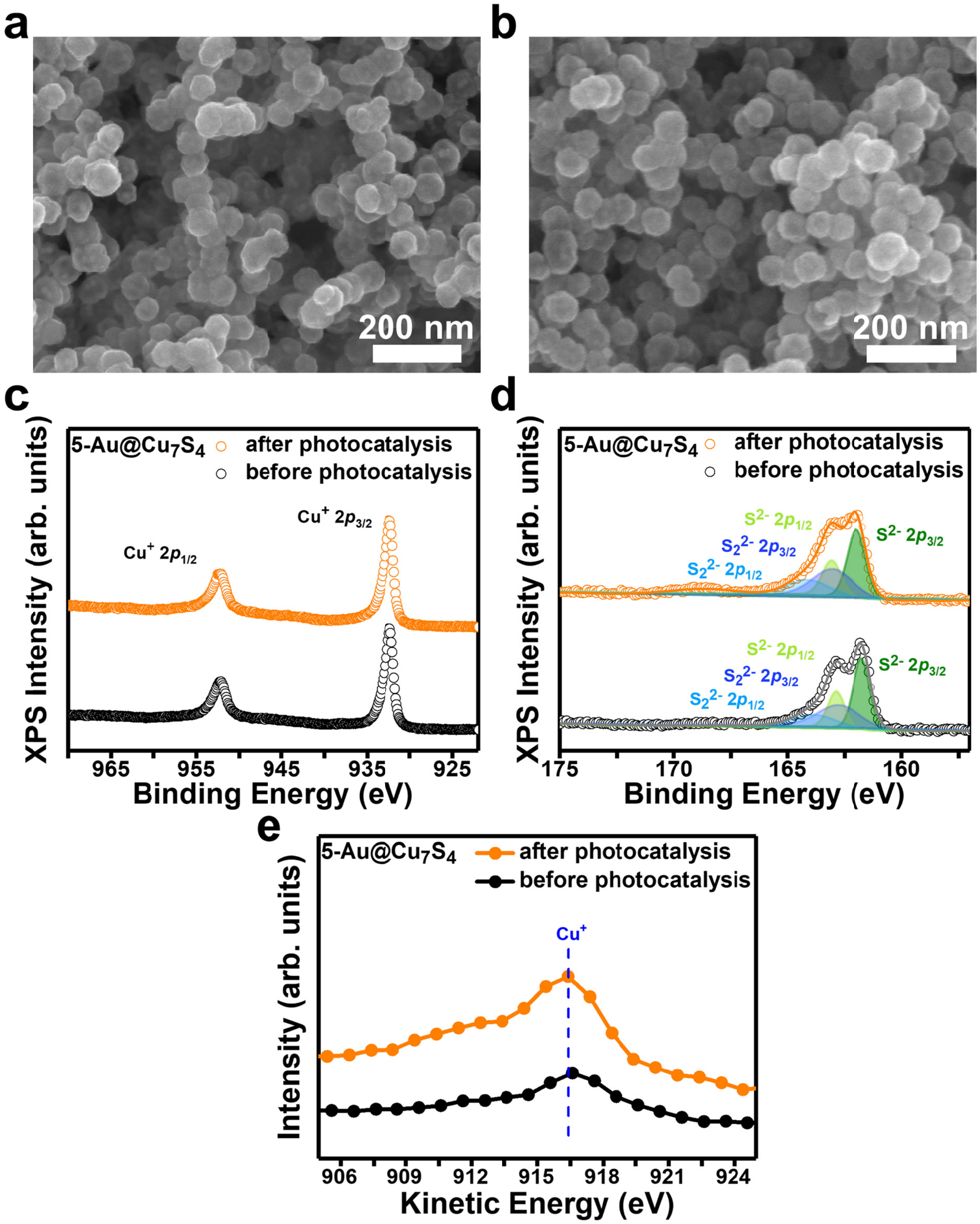

Supplementary Fig. 5 Stability tests on 5-Au@Cu_7S_4 for solar hydrogen production. SEM image of 5-Au@Cu_7S_4 **a** before and **b** after used in hydrogen production for 30 successive hours. Corresponding XPS spectra of **c** Cu 2*p*, **d** S 2*p* core levels, and **e** Auger spectra of Cu *LMM*.

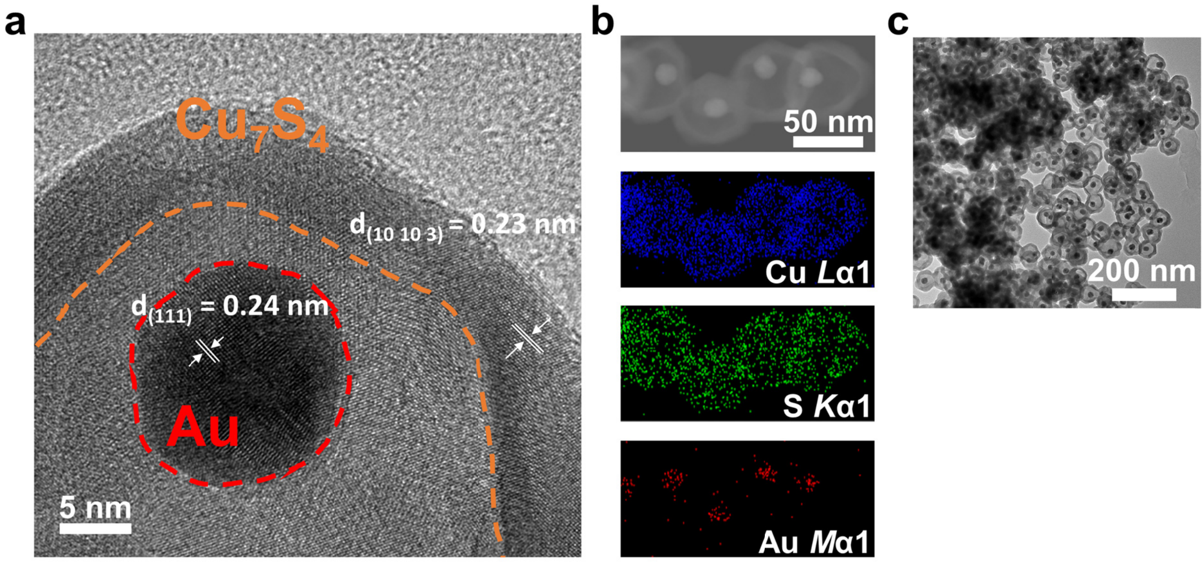

Supplementary Fig. 6 Crystallographic structure and elemental composition of the used 5-Au@Cu_7S_4. **a** HRTEM image, **b** TEM-EDS mapping profiles, **c** TEM image of 5-Au@Cu_7S_4 after used in hydrogen production for 30 successive hours.

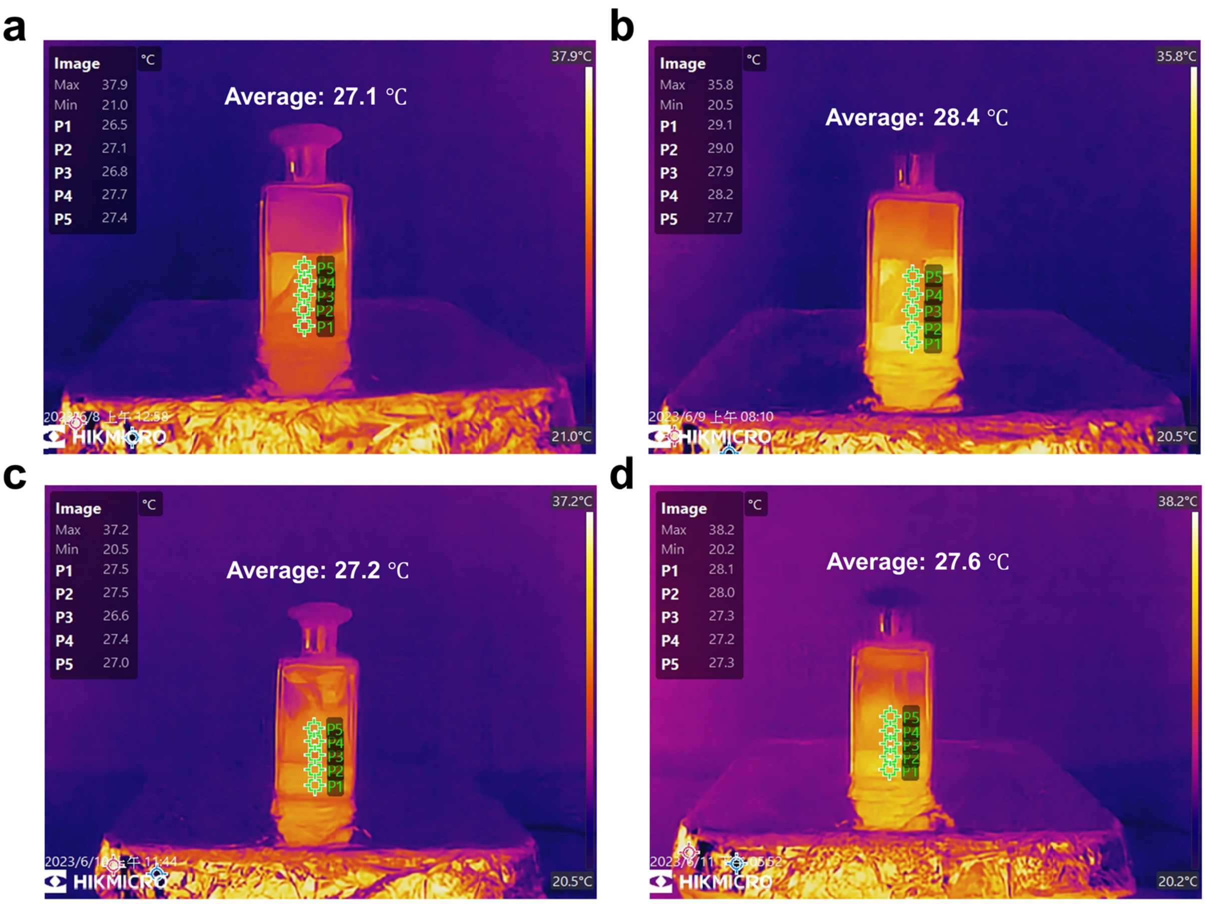

Supplementary Fig. 7 Thermal effect induced by light irradiation. Temperature profiles of the electrolyte containing 5-Au@Cu_7S_4 **a** before and **b** after for 30 successive hours of AM 1.5 G illumination. **c** and **d** show the corresponding temperature profiles of pure electrolyte. The temperature was recorded at five positions along the vertical direction of the vessel. An averaged value was then present.

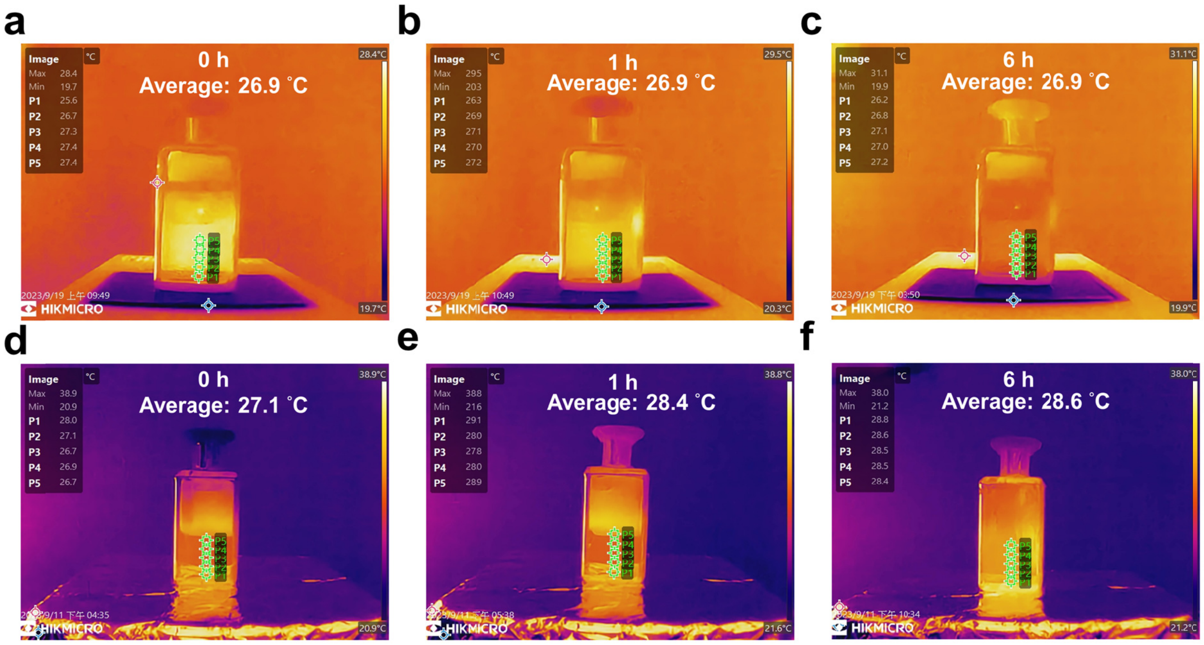

Supplementary Fig. 8 Thermal effect with and without temperature control. Temperature profiles of the electrolyte containing 5-Au@Cu_7S_4 under temperature control **a** before, **b** after for 1 hour and **c** after 6 hours of AM 1.5 G illumination. **d**, **e** and **f** show the corresponding temperature profiles of the same electrolyte without temperature control. The temperature was recorded at five positions along the vertical direction of the vessel. An averaged value was then present. The higher background temperature of **a**, **b**, **c** than that of **d**, **e**, **f** resulted from the heat generated by the cold plate under operation, which can be confirmed in **Supplementary Fig. 9**. Nevertheless, the electrolyte temperature can be well-controlled by the cold plate.

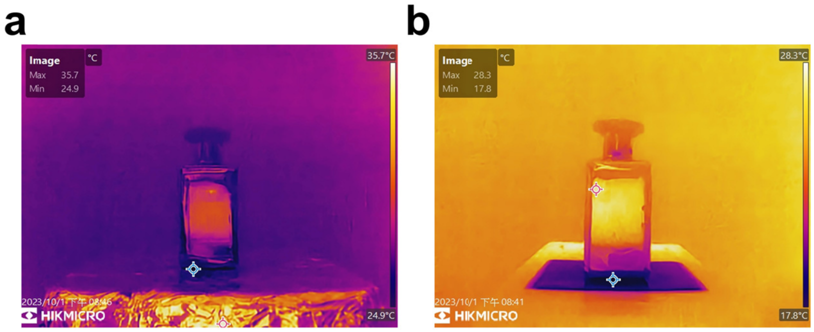

Supplementary Fig. 9 Background temperature disturbation by cold plate. Temperature profiles of pure electrolyte in dark **a** as the vessel was placed on a plain plate without temperature control, and **b** as the vessel was placed on a cold plate under temperature control. As the cold plate operated, it generated heat to cause a slight increase in background temperature.

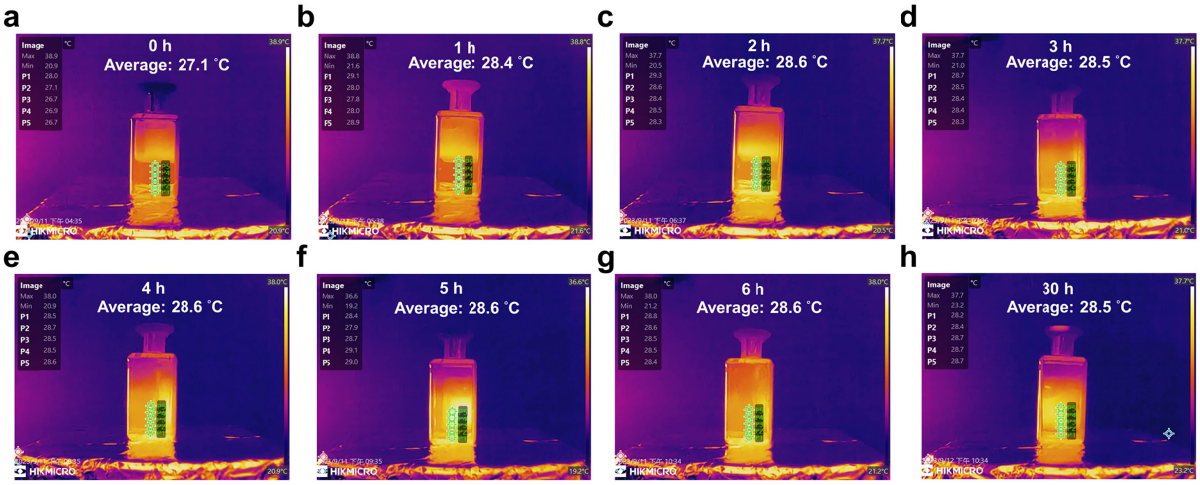

Supplementary Fig. 10 Thermal effect induced by light irradiation on Au@Cu_7S_4. Temperature profiles of the electrolyte containing 5-Au@Cu_7S_4 taken at a given time interval of AM 1.5 G illumination. The temperature was recorded at five positions along the vertical direction of the vessel. An averaged value was then present.

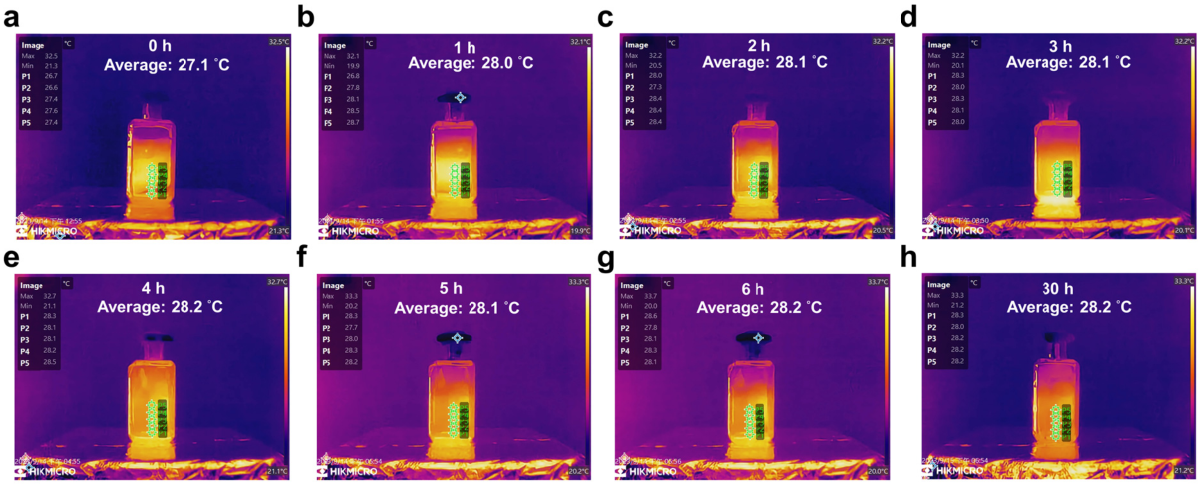

Supplementary Fig. 11 Thermal effect induced by light irradiation on pure Au. Temperature profiles of the electrolyte containing pure Au taken at a given time interval of AM 1.5 G illumination. The temperature was recorded at five positions along the vertical direction of the vessel. An averaged value was then present.

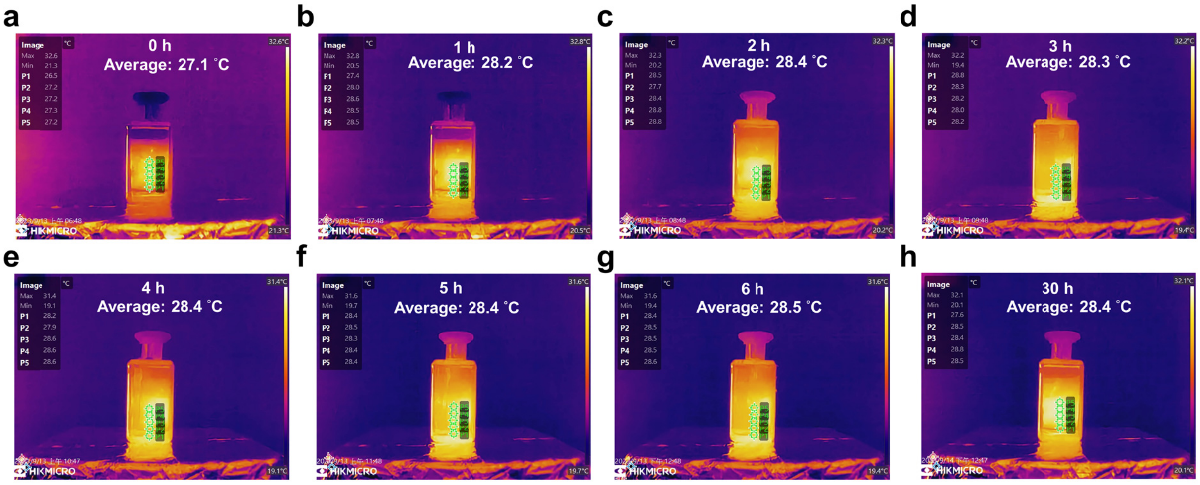

Supplementary Fig. 12 Thermal effect induced by light irradiation on pure Cu_7S_4. Temperature profiles of the electrolyte containing pure Cu_7S_4 taken at a given time interval of AM 1.5 G illumination. The temperature was recorded at five positions along the vertical direction of the vessel. An averaged value was then present.

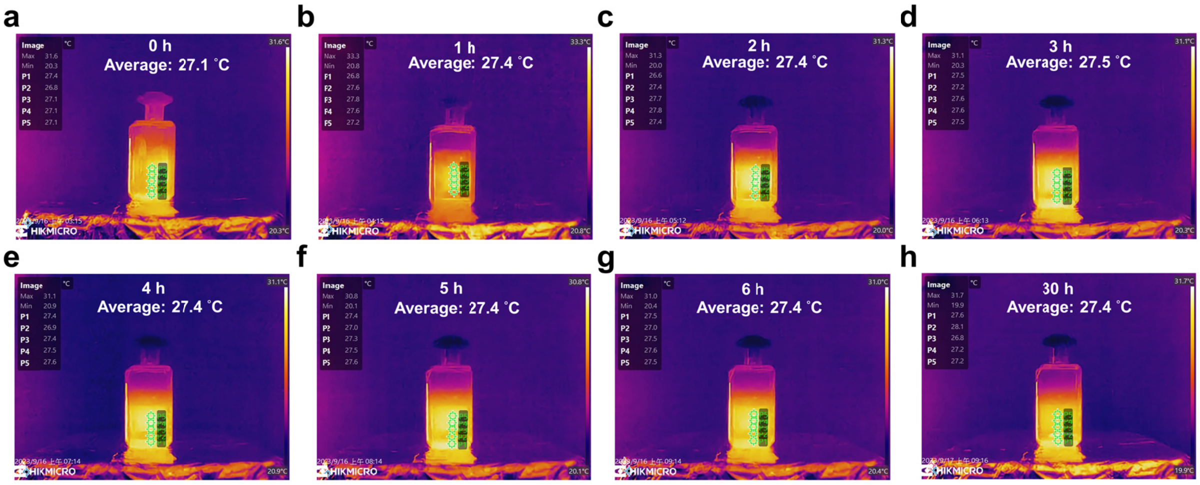

Supplementary Fig. 13 Thermal effect induced by light irradiation on pure electrolyte. Temperature profiles of pure electrolyte taken at a given time interval of AM 1.5 G illumination. The temperature was recorded at five positions along the vertical direction of the vessel. An averaged value was then present.

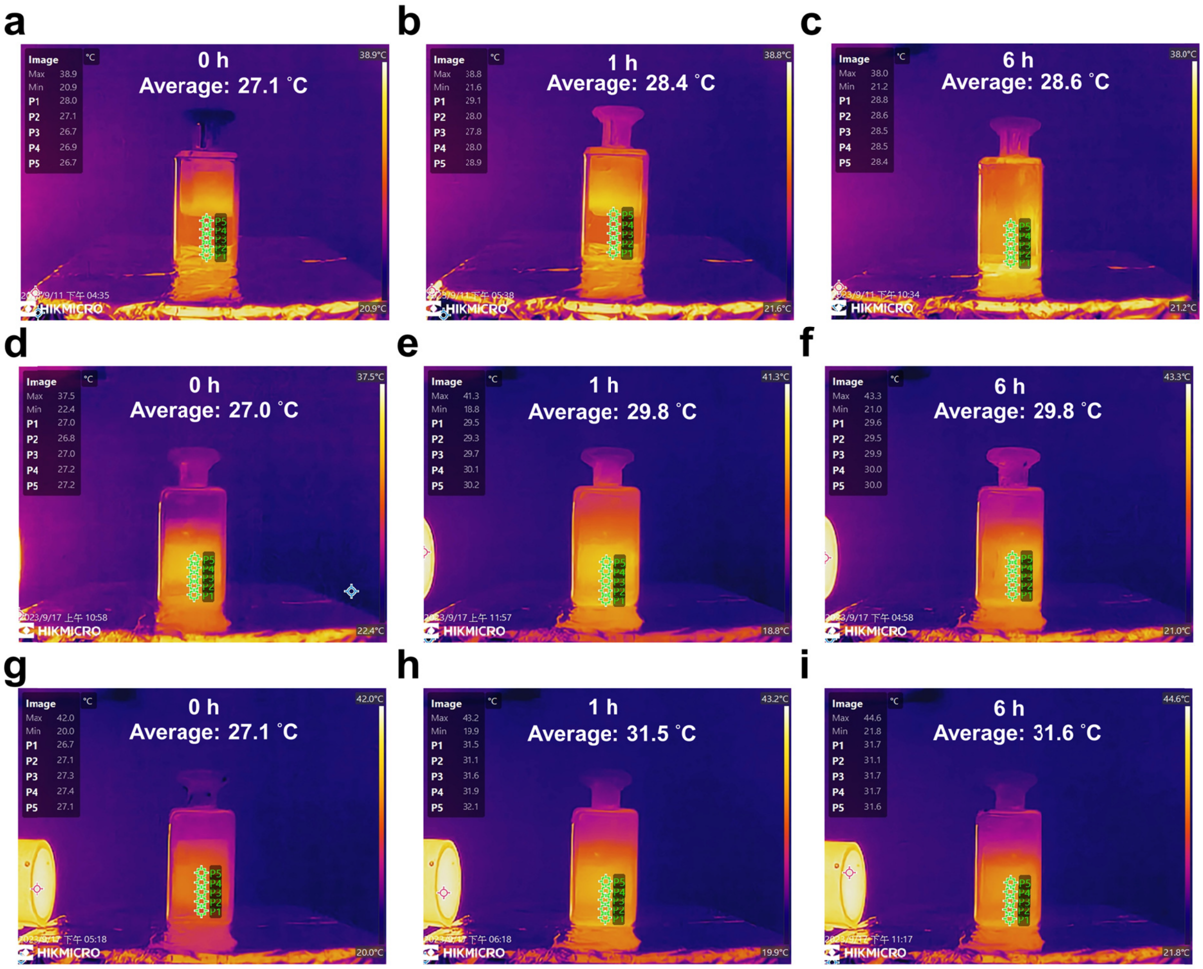

Supplementary Fig. 14 Effect of irradiation power on temperature rise for Au@Cu_7S_4. Temperature profiles of the electrolyte containing 5-Au@Cu_7S_4 taken at a given time interval under different irradiation conditions: **a**, **b**, **c** one-sun irradiation (100 mW cm^{-2}); **d**, **e**, **f** two-sun irradiation (200 mW cm^{-2}); **g**, **h**, **i** three-sun irradiation (300 mW cm^{-2}). The temperature was recorded at five positions along the vertical direction of the vessel. An averaged value was then present.

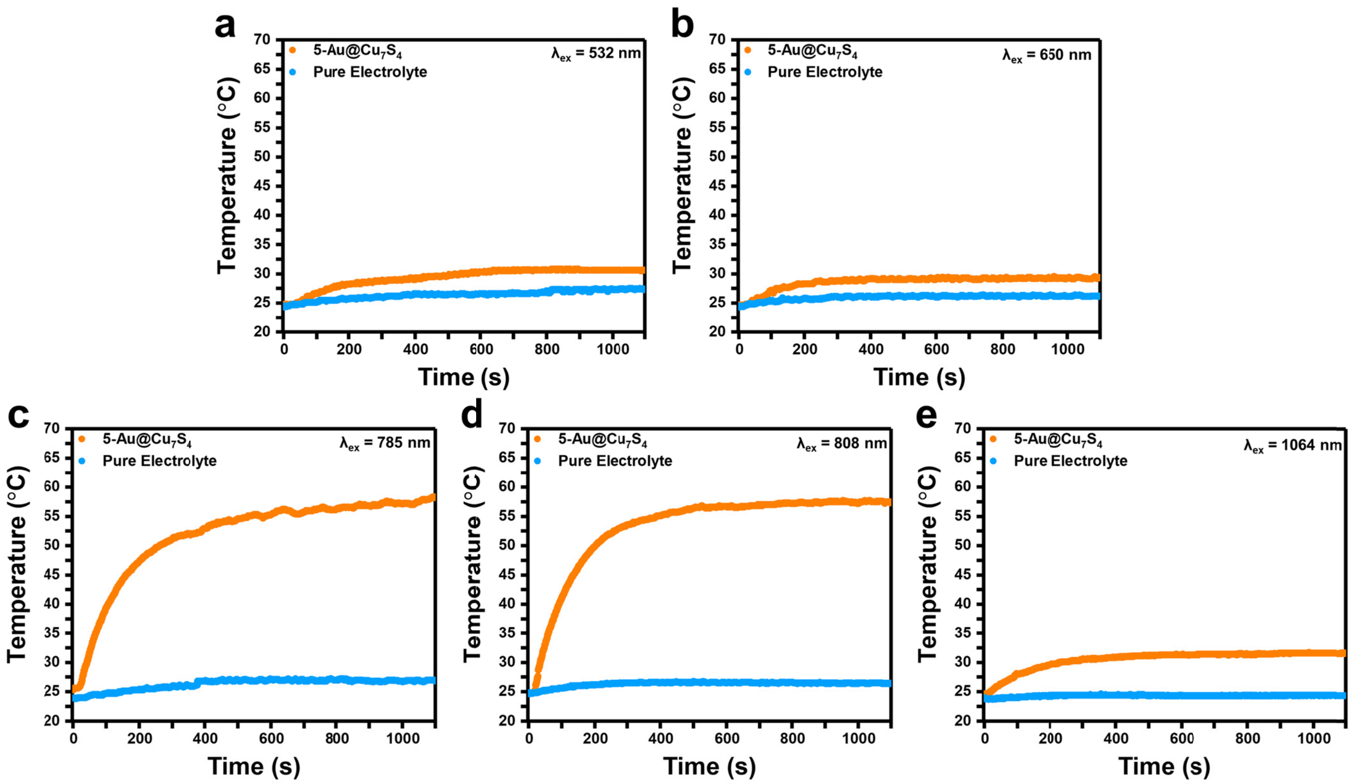

Supplementary Fig. 15 Photothermal heating upon Au@Cu_7S_4. Temperature evolution of 5-Au@Cu_7S_4-contained electrolyte (0.2 mL) under laser excitation at **a** 532 nm (2.0 W cm^{-2}), **b** 650 nm (1.0 W cm^{-2}), **c** 785 nm (4.0 W cm^{-2}), **d** 808 nm (2.0 W cm^{-2}), and **e** 1064 nm (0.33 W cm^{-2}). The power of irradiation was adjusted to the maximal capacity of the laser in order to highlight the photothermal effect. The rise of electrolyte temperature of 5-Au@Cu_7S_4 under 1064 nm irradiation was less pronounced than that recorded under 808 nm irradiation because of the lower laser power employed. The results of pure electrolyte were also included.

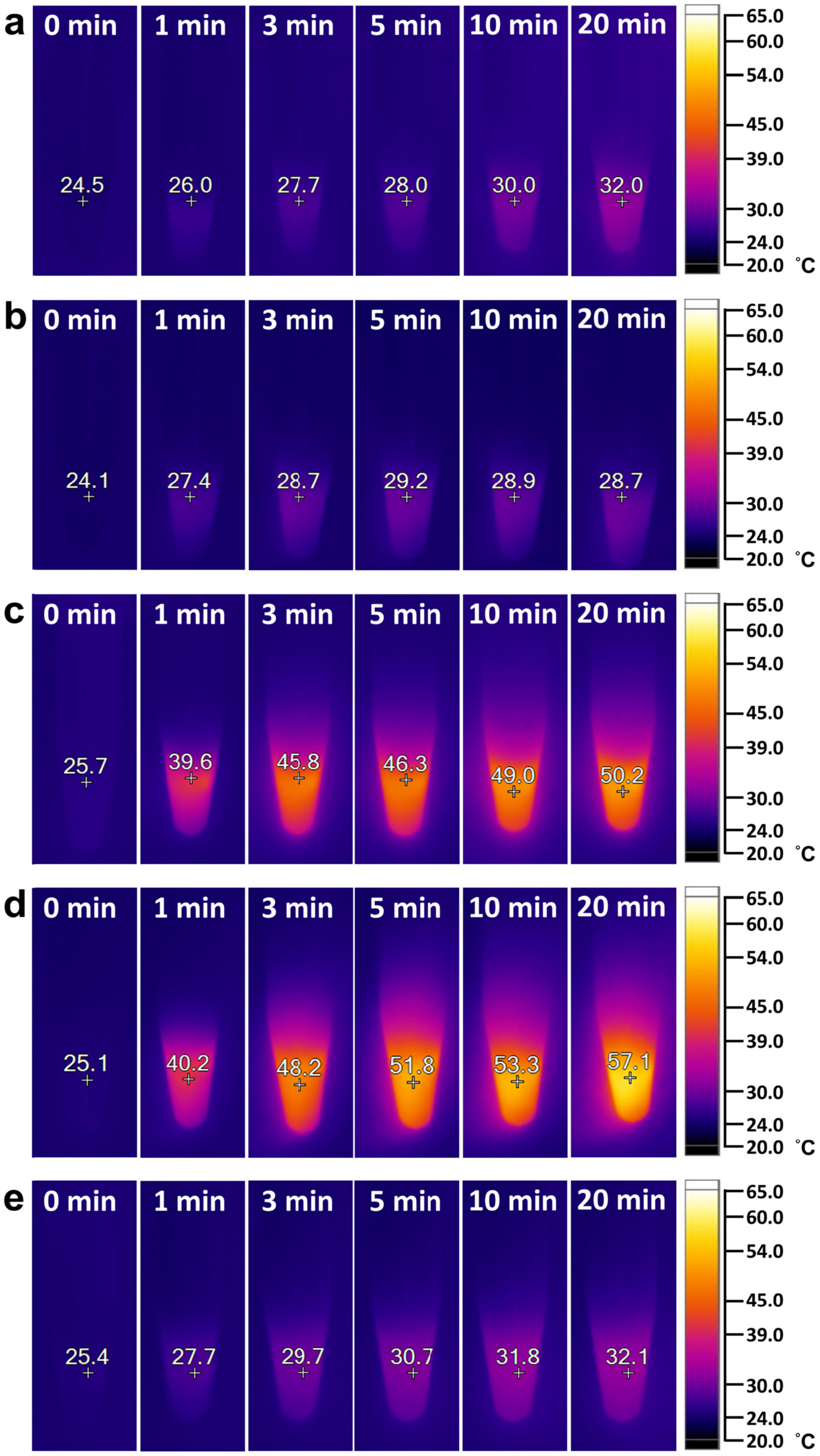

Supplementary Fig. 16 Photothermal heating upon Au@Cu_7S_4. Thermograph images of 5-Au@Cu_7S_4-contained electrolyte (volume = 0.2 mL) in a vial (capacity = 0.4 mL) taken at a given time interval under laser excitation at **a** 532 nm (2.0 W cm^{-2}), **b** 650 nm (1.0 W cm^{-2}), **c** 785 nm (4.0 W cm^{-2}), **d** 808 nm (2.0 W cm^{-2}), and **e** 1064 nm (0.33 W cm^{-2}). The power of irradiation was adjusted to the maximal capacity of the laser in order to highlight the photothermal effect.

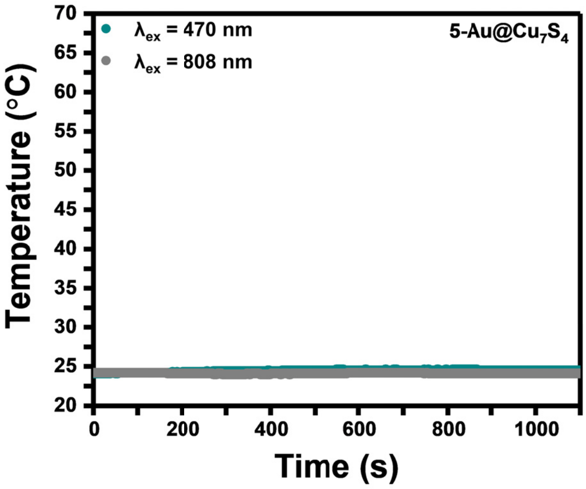

Supplementary Fig. 17 Effectiveness of AM 1.5 G illumination in photothermal heating upon Au@Cu_7S_4. Temperature evolution of 5-Au@Cu_7S_4-contained electrolyte (0.2 mL) under laser excitation at 470 nm (0.15 mW cm^{-2}) and 808 nm (0.11 mW cm^{-2}) with the power set to be equal to the irradiance of the standard AM 1.5 G spectra (ASTM G-173093 data set).

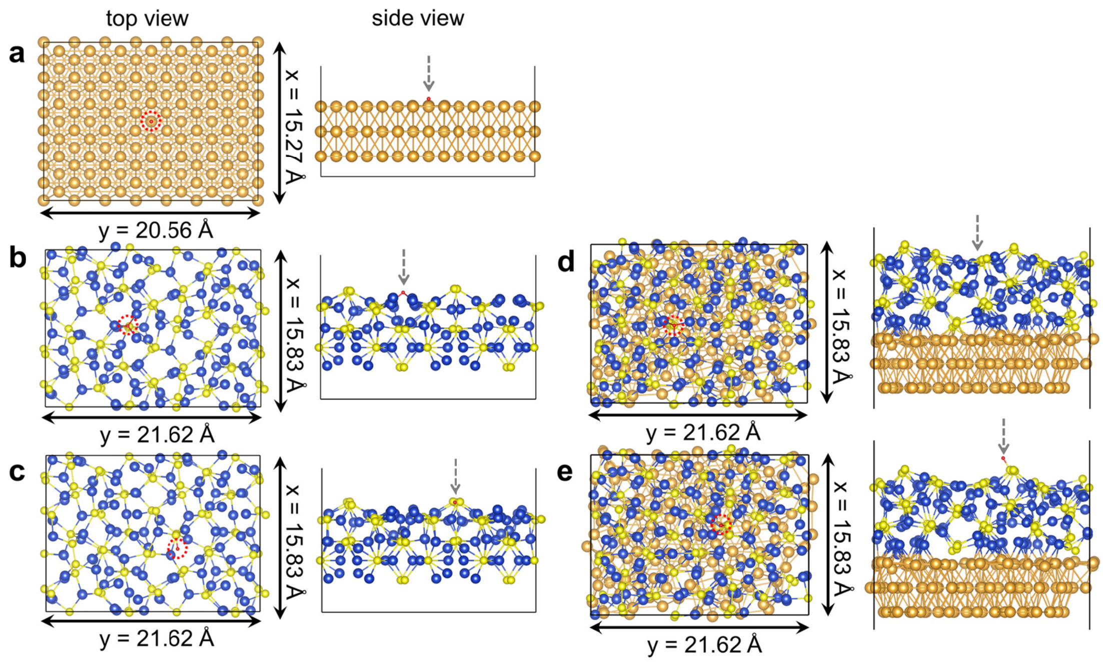

Supplementary Fig. 18 Structural models used for DFT calculations. Top and side views for various surfacial sites: **a** Au(111), **b** Cu_7S_4(010)-Cu site, **c** Cu_7S_4(010)-S site, **d** Au(111)@Cu_7S_4(010)-Cu site, and **e** Au(111)@Cu_7S_4(010)-S site. For Au(111), top and side views are directed along [111] and [2$\bar{1}\bar{1}$], respectively. For Cu_7S_4(010), top and side views are directed along [010] and [100], respectively. The optimal adsorption site of hydrogen atom for each surface models is marked by gray arrow and red circle. The blue, yellow, gold, and red spheres represent the Cu, S, Au and H atoms, respectively.

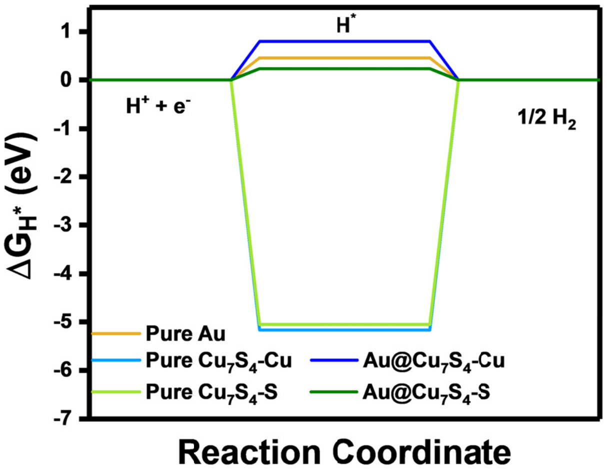

Supplementary Fig. 19 DFT calculations. $\triangle G_{H*}$ values of hydrogen adsorption at various surfacial sites, including Au site of pure Au, Cu site of pure Cu_7S_4, S site of pure Cu_7S_4, Cu site of Au@Cu_7S_4 and S site of Au@Cu_7S_4, were calculated.

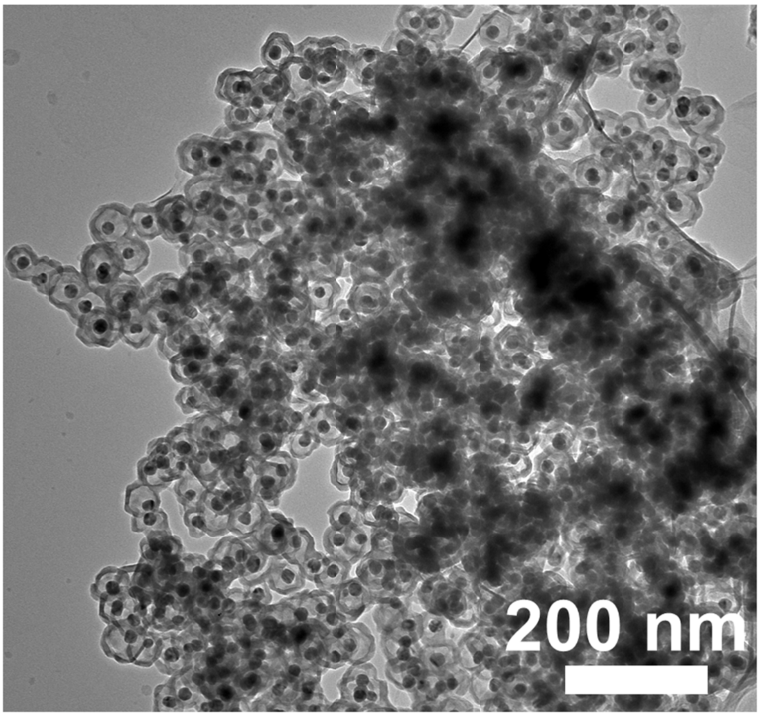

Supplementary Fig. 20 Microstructural features of 7-Au@Cu_7S_4. TEM image of 7-Au@Cu_7S_4 prepared by employing 7.0 mL of Au colloids in the synthesis of the initial Au@Cu_2O.

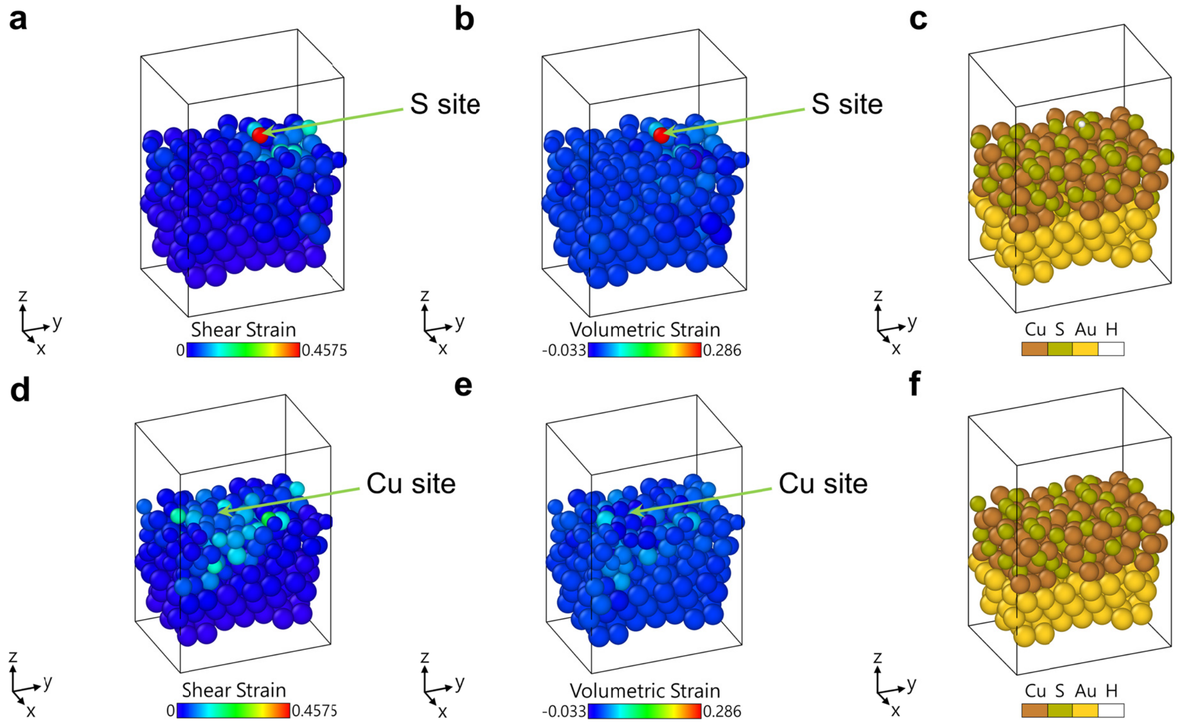

Supplementary Fig. 21 Distortion of the surface slab of Au@Cu_7S_4 upon H* adsorption. **a** Computed shear strain, **b** computed volumetric strain in the localized region where H* wad adsorbed on S site. **d** Computed shear strain, **e** computed volumetric strain in the localized region where H* wad adsorbed on Cu site. **c** and **f** show the atomic arrangement for S-H* and Cu-H* case, respectively.

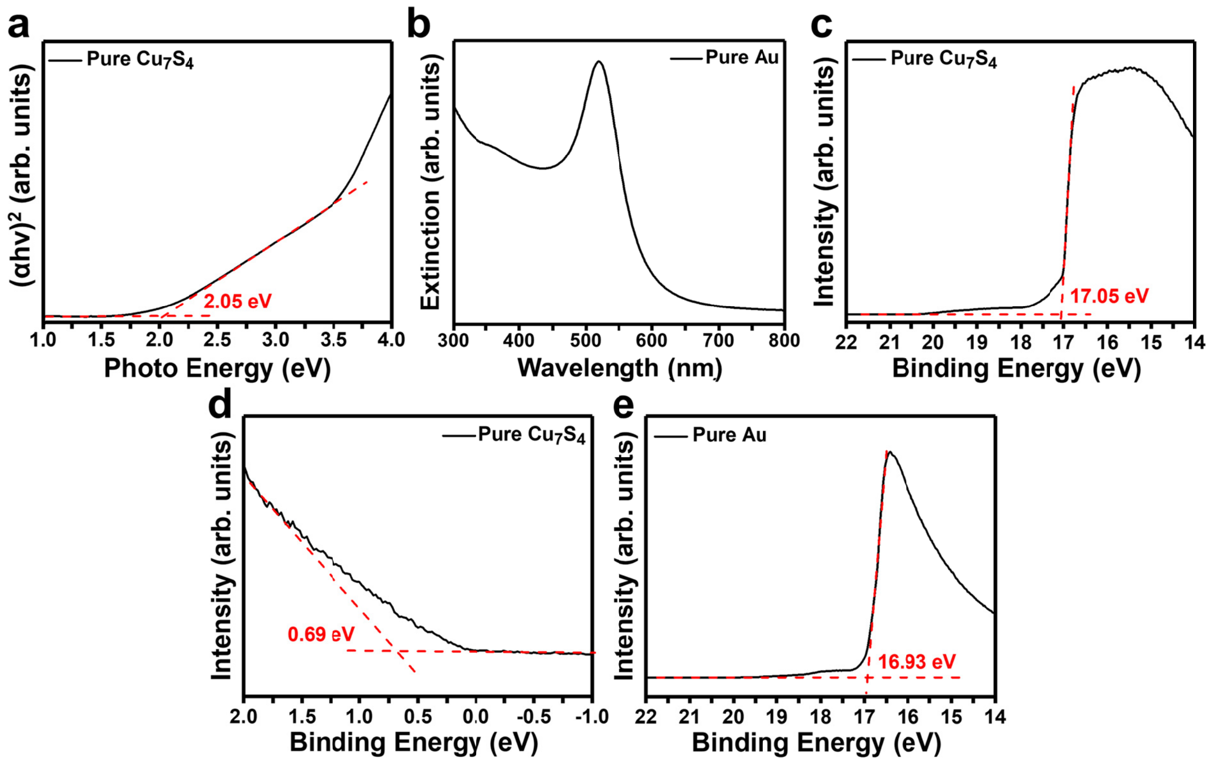

Supplementary Fig. 22 Optical properties of pure Cu_7S_4 and pure Au. **a** Tauc plot of pure Cu_7S_4. **b** UV-visible absorption spectrum of pure Au colloids. UPS spectra for **c**, **d** pure Cu_7S_4, **e** pure Au.

3. Supplementary tables

Supplementary Table 1. Fitting results of tempeoral profiles for pure Cu_7S_4 and the three Au@Cu_7S_4 under 532 nm excitaiton (Supplementary Fig. 3a). A multi-exponential function convoluted with a gaussian instrument response function was used for data fitting.

entry	A_1 (%)	τ_1 (μs)	A_2 (%)	τ_2 (μs)	A_3 (%)	τ_3 (μs)
Pure Cu_7S_4	62	0.1	38	0.7	-	-
1-Au@Cu_7S_4	61	2	35	10	4	160
3-Au@Cu_7S_4	52	2	38	10	10	160
5-Au@Cu_7S_4	49	2	44	10	7	160

Supplementary Table 2. Fitting results of tempeoral profiles for pure Cu_7S_4 and the three Au@Cu_7S_4 under 1064 nm excitaiton (Supplementary Fig. 3b). A multi-exponential function convoluted with a gaussian instrument response function was used for data fitting.

entry	A_1 (%)	τ_1 (ns)	A_2 (%)	τ_2 (μs)
Pure Cu_7S_4	100	5	-	-
1-Au@Cu_7S_4	75	5	25	6
3-Au@Cu_7S_4	88	5	12	6
5-Au@Cu_7S_4	91	5	9	6

Supplementary Table 3. Performance comparison of photocatalytic hydrogen production with the state-of-the-art NIR responsive photocatalysts reported in the literature.

Photocatalysts [co-catalyst]	Light Source	Electrolyte	Activity	Ref.
$Au@Cu_7S_4$ [none]	AM 1.5 G	methanol/glucose	211 μmol h^{-1} g^{-1} AQY = 7.3 % (2200 nm)	This work
Au/CuSe [Pt]	300 W Xe lamp (λ > 420 nm)	0.25 M Na_2S/0.35 M Na_2SO_3	4180 μmol h^{-1} g^{-1} AQY = 0.34 % (940 nm)	27
[a]UCNPs-Pt@MOF/Au [none]	AM 1.5 G	acetonitrile/[k]TEOA	280 μmol h^{-1} g^{-1} [j]AQY = 0.0005 % (980 nm)	28
g-C_3N_4/Cs_xWO_3 [3 wt% Pt]	LED lamp (365-940 nm)	[k]TEOA (10 vol.%)	3361 μmol h^{-1} g^{-1} [j]AQY = 0.007 %	29

			(980 nm)	
[b]CZS/CoP/CoO [none]	420 nm LED lamp	lactic acid (10 %)	166 mmol h^{-1} g^{-1} AQY = 0.01% (1020 nm)	30
[c]NYF@Ag_3PO_4@BP [none]	laser light (980 nm)	glycerol (50 vol.%)	146 µmol h^{-1} g^{-1} AQY = 0.077 % (980 nm)	31
[d]BE/CdS [none]	300 W Xe lamp (λ > 420 nm)	Na_2S/Na_2SO_3	40990 µmol h^{-1} g^{-1} AQY = 0.2 % (700 nm)	32
[e]Au cluster-NP/C_3N_4 [none]	300 W Xe lamp (λ > 400 nm)	methanol (20 vol.%)	230 µmol h^{-1} g^{-1} AQY = 0.3 % (900 nm)	33
[f]7%NiS-3%CuS-CN [none]	300 W Xe lamp (λ > 400 nm)	[k]TEOA (20 vol.%)	1603 µmol h^{-1} g^{-1} AQY = 0.4 %	34

			(940 nm)	
[g]5% Co_2B/CN<br>[none]	AM 1.5 G	[k]TEOA	1137 μmol h^{-1} g^{-1}<br>AQY = 0.78 %<br>(800 nm)	35
[h]BP NS/TMC<br>[0.3 wt% Pt]	Xe lamp<br>(λ > 780 nm)	methanol (20 vol.%)	205 μmol h^{-1} g^{-1}<br>AQY = 1 %<br>(880 nm)	36
Au nanorods@MoS_2-CdS<br>[none]	Xe lamp<br>(λ > 400nm)	0.3 M Na_2S/0.3 M Na_2SO_3	29.6 mmol h^{-1} g^{-1}<br>AQY = 1.06 %<br>(700 nm)	37
W_2N/C/TiO<br>[none]	AM 1.5 G	water	3.11 μmol h^{-1} g^{-1}<br>AQY = 2 %<br>(800 nm)	38
[i]BP/WS_2<br>[none]	400 W Xe<br>lamp<br>(λ > 780 nm)	[l]ETDA	3773 μmol h^{-1} g^{-1}<br>AQY = 2.06 %	39

			(780 nm)	
CdS/Cu_7S_4<br><br>[Pt]	300 W Xe<br><br>lamp<br><br>(λ > 800 nm)	0.25 M Na_2S/0.35 M Na_2SO_3	14.7 μmol h^{-1} g^{-1}<br><br>AQY = 3.8 %<br><br>(1100 nm)	40
$W_{18}O_{49}$/Au/g-C_3N_4<br><br>[1-2 wt% Pt]	300 W Xe<br><br>lamp	[k]TEOA (15 %)	3465 μmol h^{-1} g^{-1}<br><br>AQY = 9.3 %<br><br>(1200 nm)	41

[a]UCNPs denotes upconversion nanoparticles composed of $NaYF_4$: Yb, Tm, Er.
[b]CZS denotes CdZnS.
[c]NYF means β-NaYF4:Yb^{3+}, Tm^{3+}; BP means black phosphorus.
[d]BE stands for a conjugate polymer composed of B-BT-1,4-E.
[e]NP means nanoparticle.
[f]CN is C_3N_4.
[g]CN is g-C_3N_4.
[h]BP NS/TMC denotes black phosphorus nanosheets/TiO_2 mesocrystals.
[i]BP means black phosphorus.
[j]AQY value was calculated by the authors by using relevant data provided in the paper.
[k]TEOA = triethanolamine
[l]EDTA = ethylenediaminetetraacetic acid

Supplementary Table 4. Performance comparison of photocatalytic hydrogen production with the state-of-the-art sulfides-based visible light responsive photocatalysts reported in the literature.

Photocatalysts [co-catalyst]	Light Source	Electrolyte	Activity	Ref.
Au@Cu_7S_4 [none]	AM 1.5 G	methanol/glucose	211 μmol h^{-1} g^{-1} AQY = 9.4 % (500 nm)	This work
[a]MoS_2/mpg-C_3N_4 [Pt]	300W Xe lamp (λ > 420 nm)	lactic acid (10 vol.%)	969 μmol h^{-1} g^{-1} AQY = 2.1 % (420 nm)	42
[b]CoS_2/CN [none]	300W Xe lamp (λ > 420 nm)	[e]TEOA (20 vol.%)	1707 μmol h^{-1} g^{-1} AQY = 2.1 % (420 nm)	43
CuSe/CdS [none]	300W Xe lamp (λ > 420 nm)	1 M Na_2S/0.25 M Na_2SO_3	1402 μmol h^{-1} g^{-1} AQY = 3.7 %	44

			(420 nm)	
$CdS/Cu_7S_4/g\text{-}C_3N_4$<br>[none]	300W Xe lamp<br>(λ > 420 nm)<br>180 mW cm^{-2}	0.25 M Na_2S/0.35 M Na_2SO_3	3570 μmol h^{-1} g^{-1}<br>AQY = 4.4 %<br>(420 nm)	45
Cu_xS/CdS<br>[none]	300W Xe lamp<br>(λ > 420 nm)<br>470 mW	0.75 M Na_2S/1.05 M Na_2SO_3	111 mmol h^{-1} g^{-1}<br>AQY = 7 %<br>(420 nm)	46
[c]BE/CdS<br>[none]	300W Xe lamp<br>(λ > 420 nm)	Na_2S/Na_2SO_3	40990 μmol h^{-1} g^{-1}<br>AQY = 7.5 %<br>(420 nm)	32
MoS_2/TiO_2<br>[none]	LED lamp<br>(365 nm)	methanol (10 vol.%)	2443 μmol h^{-1} g^{-1}<br>AQY = 8.3 %<br>(365 nm)	47
[d]ZCSCS-30<br>[none]	5W white light	lactic acid (10 vol.%)	111 mmol h^{-1} g^{-1}<br>AQY = 8.52 %	48

			(420 nm)	
Cu_2S/CdS<br>[none]	300W Xe lamp<br>($\lambda > 420$ nm)	0.25 M Na_2S/0.35 M Na_2SO_3	2000 μmol h^{-1} g^{-1}<br>AQY = 9.5 %<br>(420 nm)	49
$CdS\text{-}Cu_{1.81}S$<br>[none]	AM 1.5 G	0.1 M Na_2S/0.1 M Na_2SO_3	2714 μmol h^{-1} g^{-1}<br>AQY = 11.32 %<br>(420 nm)	50
p-CuS/n-CdS<br>[none]	300W Xe lamp<br>($\lambda > 420$ nm)	0.25 M Na_2S/0.35 M Na_2SO_3	280 μmol h^{-1} g^{-1}<br>AQY = 12.6 %<br>(420 nm)	51
$Cd_{0.1}Cu_{0.01}Zn_{0.89}S$<br>[0.5 wt% Pt]	350W Xe lamp<br>($\lambda > 430$ nm)	0.2 M Na_2S/0.3 M Na_2SO_3	1856 μmol h^{-1} g^{-1}<br>AQY = 14.9 %<br>(420 nm)	52
$NiPS_3/CdS$<br>[none]	Xe lamp<br>($\lambda > 400$nm)	[e]TEOA (17 vol.%)	13600 μmol h^{-1} g^{-1}<br>AQY = 20.2 %	53

			(420 nm)	
ZnO/CuS [none]	AM 1.5 G	0.25 M Na_2S/0.25 M Na_2SO_3	10.11 mmol h^{-1} g^{-1} AQY = 22.3 % (420 nm)	54
Au nanorods@MoS_2-CdS [none]	Xe lamp (λ >400nm)	0.3 M Na_2S/0.3 M Na_2SO_3	29.6 mmol h^{-1} g^{-1} AQY = 35.7 % (450 nm)	37
Wood/MoS_2 [none]	AM 1.5G	deionized water	4073.3 μmol h^{-1} g^{-1} AQY = 35.9 % (420 nm)	55
TiO_2/Au@NiS_{1+x} [none]	LED lamp (365 nm)	ethanol (25 vol.%)	9616 μmol h^{-1} g^{-1} AQY = 46.0 % (365 nm)	56
$ZnIn_2S_4$ [0.26 wt% Pt]	Xe lamp (λ >420nm)	[e]TEOA (10 vol.%)	1020 μmol h^{-1} g^{-1} AQY = 50.4 %	57

			(420 nm)	
MoS_2/TiO_2 [none]	300W Xe lamp	ethanol (20 vol.%)	4300 µmol h^{-1} g^{-1} AQY = not available	58

[a]mpg-C_3N_4 means mesoporous g-C_3N_4.

[b]CN is C_3N_4.

[c]BE stands for a conjugate polymer composed of B-BT-1,4-E.

[d]ZCSCS denotes $ZnCo_2S_4$/CdS.

[e]TEOA = triethanolamine

Supplementary Table 5. Calculated specific E_{ads} and ΔE_{ZPE} corresponding to different adsorption sites.

Adsorption Site	E_{ads} (eV)	ΔE_{ZPE} (eV)	ΔG_{H*} (eV)
Au(111)-Au	0.102	0.154	0.46
Cu_7S_4(010)-Cu	−5.769	0.403	−5.17
Cu_7S_4(010)-S	−5.626	0.376	−5.05
Au(111)@Cu_7S_4(010)-Cu	0.428	0.172	0.80
Au(111)@Cu_7S_4(010)-S	−0.212	0.247	0.23

Supplementary Table 6. Coordinates of the atoms constituting the structural models of Au(111), Cu_7S_4(010) and Au(111)@Cu_7S_4(010).

Au(111)	Coordinates (Å)		
Atoms	x	y	z
Au	0	0	1.96697
Au	0	5.088447	1.96697
Au	0	10.1769	1.96697
Au	2.937816	0	1.96697
Au	2.937816	5.088447	1.96697
Au	2.937816	10.1769	1.96697
Au	5.875633	0	1.96697
Au	5.875633	5.088447	1.96697
Au	5.875633	10.1769	1.96697
Au	8.813449	0	1.96697
Au	8.813449	5.088447	1.96697
Au	8.813449	10.1769	1.96697
Au	11.75127	0	1.96697
Au	11.75127	5.088447	1.96697
Au	11.75127	10.1769	1.96697
Au	14.68908	0	1.96697
Au	14.68908	5.088447	1.96697
Au	14.68908	10.1769	1.96697
Au	17.6269	0	1.96697
Au	17.6269	5.088447	1.96697
Au	17.6269	10.1769	1.96697
Au	1.468908	2.544224	1.96697
Au	1.468908	7.632671	1.96697
Au	1.468908	12.72112	1.96697
Au	4.406725	2.544224	1.96697
Au	4.406725	7.632671	1.96697
Au	4.406725	12.72112	1.96697
Au	7.344541	2.544224	1.96697
Au	7.344541	7.632671	1.96697
Au	7.344541	12.72112	1.96697
Au	10.28236	2.544224	1.96697
Au	10.28236	7.632671	1.96697
Au	10.28236	12.72112	1.96697
Au	13.22018	2.544224	1.96697

Au	13.22018	7.632671	1.96697
Au	13.22018	12.72112	1.96697
Au	16.15799	2.544224	1.96697
Au	16.15799	7.632671	1.96697
Au	16.15799	12.72112	1.96697
Au	19.09581	2.544224	1.96697
Au	19.09581	7.632671	1.96697
Au	19.09581	12.72112	1.96697
Au	1.468908	0.84806	4.378585
Au	1.468908	5.936507	4.378585
Au	1.468908	11.02496	4.378585
Au	4.406725	0.84806	4.378585
Au	4.406725	5.936507	4.378585
Au	4.406725	11.02496	4.378585
Au	7.344541	0.84806	4.378585
Au	7.344541	5.936507	4.378585
Au	7.344541	11.02496	4.378585
Au	10.28236	0.84806	4.378585
Au	10.28236	5.936507	4.378585
Au	10.28236	11.02496	4.378585
Au	13.22018	0.84806	4.378585
Au	13.22018	5.936507	4.378585
Au	13.22018	11.02496	4.378585
Au	16.15799	0.84806	4.378585
Au	16.15799	5.936507	4.378585
Au	16.15799	11.02496	4.378585
Au	19.09581	0.84806	4.378585
Au	19.09581	5.936507	4.378585
Au	19.09581	11.02496	4.378585
Au	0	3.392284	4.378585
Au	0	8.480731	4.378585
Au	0	13.56918	4.378585
Au	2.937816	3.392284	4.378585
Au	2.937816	8.480731	4.378585
Au	2.937816	13.56918	4.378585
Au	5.875633	3.392284	4.378585
Au	5.875633	8.480731	4.378585
Au	5.875633	13.56918	4.378585
Au	8.813449	3.392284	4.378585
Au	8.813449	8.480731	4.378585

Au	8.813449	13.56918	4.378585
Au	11.75127	3.392284	4.378585
Au	11.75127	8.480731	4.378585
Au	11.75127	13.56918	4.378585
Au	14.68908	3.392284	4.378585
Au	14.68908	8.480731	4.378585
Au	14.68908	13.56918	4.378585
Au	17.6269	3.392284	4.378585
Au	17.6269	8.480731	4.378585
Au	17.6269	13.56918	4.378585
Au	0	1.696064	6.775393
Au	0	6.784511	6.790393
Au	0	11.87296	6.790393
Au	2.937816	1.696064	6.790393
Au	2.937816	6.784511	6.790393
Au	2.937816	11.87296	6.790393
Au	5.875633	1.696064	6.790393
Au	5.875633	6.784511	6.790393
Au	5.875633	11.87296	6.790393
Au	8.813449	1.696064	6.790393
Au	8.813449	6.784511	6.790393
Au	8.813449	11.87296	6.790393
Au	11.75127	1.696064	6.790393
Au	11.75127	6.784511	6.790393
Au	11.75127	11.87296	6.790393
Au	14.68908	1.696064	6.790393
Au	14.68908	6.784511	6.790393
Au	14.68908	11.87296	6.790393
Au	17.6269	1.696064	6.790393
Au	17.6269	6.784511	6.790393
Au	17.6269	11.87296	6.790393
Au	1.468908	4.240288	6.790393
Au	1.468908	9.328736	6.790393
Au	1.468908	14.41718	6.790393
Au	4.406725	4.240288	6.790393
Au	4.406725	9.328736	6.790393
Au	4.406725	14.41718	6.790393
Au	7.344541	4.240288	6.790393
Au	7.344541	9.328736	6.790393
Au	7.344541	14.41718	6.790393

Au	10.28236	4.240288	6.790393
Au	10.28236	9.328736	6.790393
Au	10.28236	14.41718	6.790393
Au	13.22018	4.240288	6.790393
Au	13.22018	9.328736	6.790393
Au	13.22018	14.41718	6.790393
Au	16.15799	4.240288	6.790393
Au	16.15799	9.328736	6.790393
Au	16.15799	14.41718	6.790393
Au	19.09581	4.240288	6.790393
Au	19.09581	9.328736	6.790393
Au	19.09581	14.41718	6.790393

Cu_7S_4(010)	Coordinates (Å)		
Atoms	x	y	z
Cu	4.059319	1.782336	9.282761
Cu	4.059319	12.590119	9.282761
Cu	11.9759	1.782336	9.282761
Cu	11.9759	12.590119	9.282761
Cu	7.806314	7.111293	5.199943
Cu	7.806314	17.919077	5.199943
Cu	15.722894	7.111293	5.199943
Cu	15.722894	17.919077	5.199943
Cu	3.892753	9.335617	9.665418
Cu	3.892753	20.143399	9.665418
Cu	11.809333	9.335617	9.665418
Cu	11.809333	20.143399	9.665418
Cu	4.017622	4.82912	9.931912
Cu	4.017622	15.636903	9.931912
Cu	11.934203	4.82912	9.931912
Cu	11.934203	15.636903	9.931912
Cu	2.197308	1.763536	3.911023
Cu	2.197308	12.571319	3.911023
Cu	10.113889	1.763536	3.911023
Cu	10.113889	12.571319	3.911023
Cu	0.101029	3.621555	9.282761
Cu	0.101029	14.429338	9.282761
Cu	8.017611	3.621555	9.282761
Cu	8.017611	14.429338	9.282761

Cu	2.684867	4.746417	3.366695
Cu	2.684867	15.554199	3.366695
Cu	10.601448	4.746417	3.366695
Cu	10.601448	15.554199	3.366695
Cu	6.570375	0.677286	7.093046
Cu	6.570375	11.485068	7.093046
Cu	14.486956	0.677286	7.093046
Cu	14.486956	11.485068	7.093046
Cu	6.721984	9.085605	10.112027
Cu	6.721984	19.893388	10.112027
Cu	14.638564	9.085605	10.112027
Cu	14.638564	19.893388	10.112027
Cu	1.711452	7.225064	7.791872
Cu	1.711452	18.032846	7.791872
Cu	9.628033	7.225064	7.791872
Cu	9.628033	18.032846	7.791872
Cu	3.84835	6.353735	5.199943
Cu	3.84835	17.161517	5.199943
Cu	11.76493	6.353735	5.199943
Cu	11.76493	17.161517	5.199943
Cu	5.232105	5.9511	7.347933
Cu	5.232105	16.758883	7.347933
Cu	13.148687	5.9511	7.347933
Cu	13.148687	16.758883	7.347933
Cu	5.669743	8.98661	7.791872
Cu	5.669743	19.794392	7.791872
Cu	13.586324	8.98661	7.791872
Cu	13.586324	19.794392	7.791872
Cu	0.059332	0.574771	9.931912
Cu	0.059332	11.382554	9.931912
Cu	7.975912	0.574771	9.931912
Cu	7.975912	11.382554	9.931912
Cu	1.242119	8.990669	9.717354
Cu	1.242119	19.798453	9.717354
Cu	9.1587	8.990669	9.717354
Cu	9.1587	19.798453	9.717354
Cu	2.763693	7.126069	10.112027
Cu	2.763693	17.933851	10.112027
Cu	10.680274	7.126069	10.112027
Cu	10.680274	17.933851	10.112027

Cu	7.851043	6.876055	9.665418
Cu	7.851043	17.683837	9.665418
Cu	15.767625	6.876055	9.665418
Cu	15.767625	17.683837	9.665418
Cu	2.612086	4.726605	7.093046
Cu	2.612086	15.534388	7.093046
Cu	10.528666	4.726605	7.093046
Cu	10.528666	15.534388	7.093046
Cu	6.643157	0.657475	3.366695
Cu	6.643157	11.465257	3.366695
Cu	14.559737	0.657475	3.366695
Cu	14.559737	11.465257	3.366695
Cu	3.848024	9.10038	5.199943
Cu	3.848024	19.908162	5.199943
Cu	11.764604	9.10038	5.199943
Cu	11.764604	19.908162	5.199943
Cu	4.587287	1.866531	5.199943
Cu	4.587287	12.674312	5.199943
Cu	12.503867	1.866531	5.199943
Cu	12.503867	12.674312	5.199943
Cu	6.155598	3.640355	3.911023
Cu	6.155598	14.448138	3.911023
Cu	14.072178	3.640355	3.911023
Cu	14.072178	14.448138	3.911023
Cu	2.171797	1.770734	6.440424
Cu	2.171797	12.578517	6.440424
Cu	10.088378	1.770734	6.440424
Cu	10.088378	12.578517	6.440424
Cu	6.130088	3.633157	6.440424
Cu	6.130088	14.44094	6.440424
Cu	14.046668	3.633157	6.440424
Cu	14.046668	14.44094	6.440424
Cu	5.20041	7.221004	9.717354
Cu	5.20041	18.028786	9.717354
Cu	13.11699	7.221004	9.717354
Cu	13.11699	18.028786	9.717354
Cu	7.80664	9.857939	5.199943
Cu	7.80664	20.66572	5.199943
Cu	15.723221	9.857939	5.199943
Cu	15.723221	20.66572	5.199943

Cu	1.273815	10.260573	7.347933
Cu	1.273815	21.068354	7.347933
Cu	9.190395	10.260573	7.347933
Cu	9.190395	21.068354	7.347933
Cu	0.628997	3.537361	5.199943
Cu	0.628997	14.345143	5.199943
Cu	8.545578	3.537361	5.199943
Cu	8.545578	14.345143	5.199943
S	1.668884	5.564122	5.199943
S	1.668884	16.371905	5.199943
S	9.585465	5.564122	5.199943
S	9.585465	16.371905	5.199943
S	7.887712	2.426093	11.06357
S	7.887712	13.233875	11.06357
S	15.804294	2.426093	11.06357
S	15.804294	13.233875	11.06357
S	6.188826	5.280578	9.286794
S	6.188826	16.088359	9.286794
S	14.105407	5.280578	9.286794
S	14.105407	16.088359	9.286794
S	3.999892	2.906211	7.185694
S	3.999892	13.713994	7.185694
S	11.916473	2.906211	7.185694
S	11.916473	13.713994	7.185694
S	3.859548	7.818937	7.101452
S	3.859548	18.626719	7.101452
S	11.776129	7.818937	7.101452
S	11.776129	18.626719	7.101452
S	5.790904	0.122605	9.199638
S	5.790904	10.930388	9.199638
S	13.707485	0.122605	9.199638
S	13.707485	10.930388	9.199638
S	7.84313	8.306307	3.214252
S	7.84313	19.114089	3.214252
S	15.759711	8.306307	3.214252
S	15.759711	19.114089	3.214252
S	1.832615	5.281286	9.199638
S	1.832615	16.089068	9.199638
S	9.749195	5.281286	9.199638
S	9.749195	16.089068	9.199638

S	7.817838	8.392736	7.101452
S	7.817838	19.200517	7.101452
S	15.734419	8.392736	7.101452
S	15.734419	19.200517	7.101452
S	3.929423	2.977797	11.06357
S	3.929423	13.785581	11.06357
S	11.846002	2.977797	11.06357
S	11.846002	13.785581	11.06357
S	2.085596	10.636959	5.184943
S	2.085596	21.444742	5.199943
S	10.002177	10.636959	5.199943
S	10.002177	21.444742	5.199943
S	0.041602	2.49768	7.185694
S	0.041602	13.305462	7.185694
S	7.958183	2.49768	7.185694
S	7.958183	13.305462	7.185694
S	2.230536	0.123313	9.286794
S	2.230536	10.931095	9.286794
S	10.147117	0.123313	9.286794
S	10.147117	10.931095	9.286794
S	3.884839	7.905366	3.214252
S	3.884839	18.713148	3.214252
S	11.801421	7.905366	3.214252
S	11.801421	18.713148	3.214252
S	6.043886	5.574714	5.199943
S	6.043886	16.382495	5.199943
S	13.960467	5.574714	5.199943
S	13.960467	16.382495	5.199943
S	5.627174	10.647552	5.199943
S	5.627174	21.455334	5.199943
S	13.543756	10.647552	5.199943
S	13.543756	21.455334	5.199943

Au(111)@Cu_7S_4(010)	Coordinates (Å)		
Atoms	x	y	z
Cu	4.551911	1.343007	17.412482
Cu	4.202546	12.331765	17.495359
Cu	12.100204	2.560924	17.407057
Cu	12.555309	13.044446	17.257728

Cu	7.675662	6.775357	13.217975
Cu	9.270526	17.868183	12.267991
Cu	0.19339	6.949001	13.549849
Cu	0.198133	16.348476	11.842364
Cu	3.614653	9.877798	16.856604
Cu	3.26075	20.499654	17.319701
Cu	11.882498	10.467762	17.036191
Cu	12.178261	21.073865	16.643374
Cu	4.672048	4.481884	17.58838
Cu	4.283963	15.507814	17.657206
Cu	12.194503	5.063643	18.622021
Cu	12.25385	15.734356	17.97793
Cu	1.603186	2.232664	11.996421
Cu	3.176063	12.730728	12.031311
Cu	8.80531	2.239793	11.667848
Cu	9.916723	12.526844	11.954639
Cu	0.253957	4.391138	17.858242
Cu	0.359259	14.728502	17.469147
Cu	8.130823	4.719572	17.79056
Cu	8.129706	15.345835	17.322395
Cu	2.841296	4.49819	12.020247
Cu	3.054861	15.233941	11.941115
Cu	11.076526	4.655251	11.921399
Cu	11.072534	14.780503	12.017731
Cu	6.491583	1.459487	15.569526
Cu	6.34362	11.761915	15.82599
Cu	14.24632	1.278392	15.822427
Cu	14.371342	11.612998	15.904122
Cu	5.571918	8.345859	17.691928
Cu	7.13158	20.695681	16.787239
Cu	13.925747	8.690172	17.74734
Cu	14.287438	19.368314	17.229321
Cu	1.825575	9.485606	14.623008
Cu	2.037722	19.225672	13.785635
Cu	9.83071	6.873014	15.419425
Cu	11.087528	17.957331	14.21873
Cu	3.723799	6.583131	13.268453
Cu	5.19899	17.758417	12.262154
Cu	12.107475	6.803788	13.101542
Cu	14.154917	18.527758	11.807041

Cu	5.839125	4.022635	15.279993
Cu	4.890498	17.144677	15.603842
Cu	14.29708	5.18803	16.151452
Cu	14.023387	16.753009	15.275744
Cu	5.879465	9.139695	15.093326
Cu	4.966658	21.077256	15.545453
Cu	13.634179	9.209822	15.256597
Cu	14.389445	19.371511	14.337807
Cu	0.008644	1.706085	17.82446
Cu	0.00032	12.077451	18.171793
Cu	8.464047	1.549608	17.3668
Cu	8.123501	12.073558	17.735103
Cu	1.016381	9.601874	17.039991
Cu	0.604362	20.832066	16.988402
Cu	7.68478	9.680687	16.822202
Cu	9.631217	20.840554	16.63321
Cu	2.865097	7.350417	16.974266
Cu	2.950791	17.889459	17.094818
Cu	9.755551	8.08795	17.776972
Cu	9.697527	18.201179	16.499007
Cu	7.493759	7.001984	16.568779
Cu	7.273882	17.924505	16.102404
Cu	0.323406	7.002745	17.22426
Cu	0.434256	17.309269	16.839969
Cu	2.461313	4.846628	16.275382
Cu	2.272324	15.73337	15.850352
Cu	10.667218	4.849303	16.656649
Cu	10.606848	15.870915	15.707068
Cu	6.204477	1.567487	11.819723
Cu	5.633685	12.440877	11.868145
Cu	14.269188	2.592138	11.851197
Cu	14.09217	11.912834	11.803953
Cu	3.934183	10.39125	12.727695
Cu	3.932093	0.238784	12.350239
Cu	11.532156	10.457844	12.009969
Cu	11.999178	21.389017	13.072449
Cu	4.084262	2.451608	10.894818
Cu	4.616044	12.699111	14.127402
Cu	11.369559	2.010606	11.910851
Cu	12.139618	12.72173	13.296076

Cu	5.63187	4.103874	12.073824
Cu	5.546235	15.069769	12.072806
Cu	13.594338	5.030848	11.821214
Cu	13.675157	14.456747	12.114275
Cu	1.600179	3.138855	14.38791
Cu	2.015387	13.76443	14.02967
Cu	9.826845	3.234239	13.846157
Cu	9.830524	12.576863	14.609117
Cu	7.307525	2.822577	13.595095
Cu	5.956393	14.488839	15.343321
Cu	13.765424	3.493939	14.173192
Cu	13.72176	13.95954	14.948845
Cu	4.99912	6.388958	15.917566
Cu	5.418234	18.891784	17.679601
Cu	12.220973	7.040989	16.671268
Cu	12.193228	18.10864	16.487447
Cu	7.817995	11.065888	12.099589
Cu	8.010206	21.481995	12.786543
Cu	15.802091	10.307227	12.714323
Cu	15.795513	0.380011	12.866807
Cu	1.944894	11.805302	15.841049
Cu	2.371473	1.094484	16.010404
Cu	9.952542	9.767896	15.435074
Cu	10.55409	1.394243	15.647093
Cu	0.240638	4.474736	12.369157
Cu	15.655613	15.192918	14.040291
Cu	8.145423	4.6071	11.982164
Cu	8.13634	15.632896	14.545758
S	1.866885	5.37303	13.904874
S	1.910255	16.107577	13.600953
S	9.691722	5.498395	13.457437
S	10.051285	16.180963	13.441241
S	15.093449	3.139158	19.445852
S	15.172439	13.924609	19.329262
S	7.445881	2.876908	18.999459
S	7.25524	13.804488	18.909691
S	6.086235	6.21186	18.080314
S	6.163657	16.813322	17.643683
S	13.931158	6.482379	18.015163
S	14.030681	17.151648	17.47955

S	3.890102	3.036793	15.989872
S	3.765084	13.849941	15.820736
S	11.687386	3.423288	15.343873
S	11.571609	13.779648	15.393167
S	3.770874	8.300633	15.164873
S	3.694107	19.168568	15.433541
S	11.650894	8.225994	14.889631
S	12.232876	19.752474	14.755711
S	5.486309	21.019876	18.212456
S	5.789599	10.56572	17.801775
S	14.207837	21.536782	17.641914
S	13.837759	10.871905	17.992939
S	7.816518	7.846239	11.220464
S	7.970214	19.49891	11.642141
S	0.199625	8.296222	11.711058
S	0.125458	19.758183	12.539853
S	1.960801	5.828798	18.280563
S	2.039499	16.183275	18.147767
S	10.000967	5.974411	18.414013
S	10.139181	16.515201	17.831745
S	8.029856	8.330685	14.886101
S	8.433711	19.617501	15.275161
S	15.792036	8.398812	15.459774
S	0.45253	19.149601	15.508293
S	5.398098	2.835366	19.012014
S	5.202526	13.807084	18.964877
S	12.988285	3.131815	19.3734
S	13.077561	14.044128	19.175973
S	1.873248	11.433246	13.443981
S	2.052706	0.929307	13.768465
S	9.690475	10.757027	13.335701
S	9.979213	0.916552	13.397291
S	15.583094	3.223428	15.903779
S	15.82209	13.507057	15.593988
S	7.949768	3.184154	15.798458
S	8.013228	13.516451	15.76211
S	1.984366	0.538569	18.165362
S	2.112125	11.220305	18.083606
S	10.575827	0.7123	17.891456
S	9.740309	10.354079	17.718405

S	3.685585	8.340328	11.854842
S	3.816243	19.494867	12.380692
S	11.719574	8.23088	11.386667
S	12.581821	20.159326	11.232557
S	5.752698	5.644467	13.703157
S	6.088286	16.361352	13.812407
S	14.027572	5.781332	13.858115
S	13.983256	16.499006	13.058771
S	6.022388	10.996751	13.604365
S	5.924059	0.362436	13.688081
S	13.746077	10.960325	13.763184
S	13.811148	1.202957	13.570889
Au	15.438311	1.249666	5.146704
Au	15.765327	3.891316	5.146704
Au	0.008976	6.601861	5.146704
Au	0.140376	9.310312	5.146704
Au	0.150536	12.063012	5.146704
Au	15.724462	14.846991	5.146704
Au	15.317057	17.668152	5.146704
Au	4.957644	1.052181	5.146704
Au	5.048964	3.794655	5.146704
Au	5.308562	6.59277	5.146704
Au	5.43389	9.412674	5.146704
Au	5.351996	12.255867	5.146704
Au	5.009799	15.054448	5.146704
Au	4.669293	17.886481	5.146704
Au	10.035469	0.949317	5.146704
Au	10.280976	3.656524	5.146704
Au	10.483428	6.433347	5.146704
Au	10.593742	9.239244	5.146704
Au	10.619362	12.069135	5.146704
Au	10.383626	14.894914	5.146704
Au	9.918198	17.685161	5.146704
Au	2.425091	2.426391	5.146704
Au	2.587042	5.242185	5.146704
Au	2.77696	8.031837	5.146704
Au	2.823406	10.814598	5.146704
Au	2.598157	13.588933	5.146704
Au	2.266462	16.348626	5.146704
Au	1.991702	19.058672	5.146704

Au	7.557033	2.273689	5.146704
Au	7.769314	5.088322	5.146704
Au	7.961792	7.939177	5.146704
Au	8.029227	10.781522	5.146704
Au	7.870089	13.590794	5.146704
Au	7.496343	16.312165	5.146704
Au	7.210898	19.036246	5.146704
Au	12.654186	1.926252	5.146704
Au	13.016093	4.710303	5.146704
Au	13.150522	7.53372	5.146704
Au	13.276913	10.315943	5.146704
Au	13.287422	13.075161	5.146704
Au	13.056142	15.805738	5.146704
Au	12.559969	18.464617	5.146704
Au	1.172987	1.941572	7.55819
Au	1.023852	4.904864	7.55819
Au	1.080705	7.786334	7.55819
Au	1.207553	10.639048	7.55819
Au	0.948167	13.577872	7.55819
Au	0.630074	16.614118	7.55819
Au	0.736536	19.768051	7.55819
Au	6.651357	1.061377	7.55819
Au	5.972035	4.80129	7.55819
Au	6.339657	7.893203	7.55819
Au	6.351866	10.95924	7.55819
Au	6.202884	14.045635	7.55819
Au	5.894895	17.025279	7.55819
Au	5.863951	19.938187	7.55819
Au	11.726654	0.58567	7.55819
Au	11.310478	4.892363	7.55819
Au	11.385787	7.831751	7.55819
Au	11.593332	10.732203	7.55819
Au	11.407801	13.647824	7.55819
Au	11.741255	16.615682	7.55819
Au	11.157884	19.363721	7.55819
Au	3.274499	21.221644	7.55819
Au	4.082052	2.373522	7.55819
Au	3.662041	6.458089	7.55819
Au	3.824614	9.409709	7.55819
Au	3.714025	12.433709	7.55819

Au	3.431791	15.395692	7.55819
Au	3.202073	18.346195	7.55819
Au	9.088124	21.252359	7.55819
Au	8.505841	3.383456	7.55819
Au	8.79235	6.340364	7.55819
Au	8.916854	9.393524	7.55819
Au	8.83392	12.399665	7.55819
Au	8.833919	15.40638	7.55819
Au	8.473922	18.329853	7.55819
Au	14.567742	0.160657	7.55819
Au	13.714889	2.972884	7.55819
Au	14.10083	5.870624	7.55819
Au	14.179606	8.725388	7.55819
Au	14.341233	11.582942	7.55819
Au	14.067148	14.550797	7.55819
Au	13.940213	18.834237	7.55819
Au	2.372833	0.483906	10.11201
Au	1.979539	3.411393	9.727763
Au	1.868593	6.230568	9.924643
Au	2.100687	9.128124	9.980411
Au	1.936616	12.250308	9.838682
Au	1.49383	15.047795	9.870514
Au	1.497584	17.800319	9.973551
Au	7.883531	0.335356	10.025673
Au	6.890702	3.117272	9.884418
Au	6.988214	5.998086	9.897441
Au	7.043411	9.743475	9.93577
Au	7.229821	12.591257	9.900022
Au	7.047974	15.461382	9.891394
Au	6.632191	18.286786	9.944391
Au	12.918024	0.678087	10.092824
Au	12.371818	3.437471	9.970257
Au	12.235816	6.256547	9.876885
Au	12.901269	9.843995	9.842987
Au	12.09859	12.639835	10.378515
Au	12.457852	15.311421	9.854102
Au	12.148398	18.134257	9.968219
Au	5.112706	0.319937	9.735035
Au	4.365676	4.872671	9.861293
Au	4.790953	7.845499	9.803525

Au	4.4572	10.965607	9.932623
Au	4.442972	13.889512	10.003462
Au	4.226279	16.693284	9.987373
Au	3.670023	19.5153	10.000284
Au	10.161482	1.813613	9.427344
Au	9.595052	4.635883	9.782714
Au	9.796872	7.803046	9.816821
Au	9.787814	10.942796	9.80666
Au	9.638163	14.032838	9.89269
Au	9.633881	16.798664	9.842106
Au	9.64131	19.640482	9.871472
Au	15.623125	1.422801	9.980057
Au	15.113392	4.245534	9.830125
Au	14.960639	7.15751	9.861894
Au	15.527154	10.654751	9.96542
Au	14.953358	13.493582	9.974907
Au	14.691871	16.956953	9.63246
Au	0.107547	20.224909	10.12068

Supplementary Table 7. Comparison of photocatalytic performance and reaction scenarios with dual-plasmonic metal-semiconductor photocatalysts reported in the literature.

Photocatalyst Composition<br>[Heterostructure Type]<br>[Co-catalyst]	Reaction Scenario	Active Region	Activity	Ref.
Au@Cu_7S_4<br>[yolk@shell]<br>[none]	photocatalytic hydrogen production	AM 1.5 G	yield = 211 μmol h^{-1} g^{-1}<br>AQY = 9.4 % at 500 nm, 2.7 % at 900 nm, 7.3 % at 2200 nm	This work
Au/CuSe<br>[tangential]<br>[Pt]	photocatalytic hydrogen production	λ > 420 nm	yield = 4180 μmol h^{-1} g^{-1}<br>AQY = 0.30 % at 500nm, 0.34 % at 940 nm	27
Au-Cu_{2-x}Te<br>[disk-on-dot]<br>[none]	photoelectrochemical hydrogen production	white light	photocurrent = 2.37 mA cm^{-2} at -0.4 V_{RHE}<br>AQY = not available	59
Au@Cu_{2-x}Se<br>[eccentric]	photocatalytic degradation of	λ > 420 nm	rate constant = 0.23 min^{-1} at λ > 420 nm, 0.13 min^{-1} at λ > 760 nm	60

[none]	rhodamine B		AQY = not available	
Au@CuS<br>[core@shell]<br>[none]	photocatalytic degradation of rhodamine B	visible to NIR	rate constant = 0.012 min^{-1} at 445 nm, 0.009 min^{-1} at 638 nm, 0.008 min^{-1} at 980 nm<br>AQY = not available	19
Au@WO_{3-x}<br>[core@shell]<br>[none]	photocatalytic hydrolysis of ammonia borane	λ > 420 nm	[a]yield = 283 μmol h^{-1} g^{-1}<br>AQY = not available	61
Au/CdS-$Cu_{2-x}S$<br>[core/shell]<br>[none]	photocatalytic degradation of rhodamine B	λ > 420 nm	rate constant = not available<br>[b]AQY = 1.68 × 10^{-12} mol mW^{-1} h^{-1}	23
Au/$Cu_{2-x}S$<br>[half-shell]<br>[none]	photocatalytic degradation of methylene blue	λ > 420 nm	rate constant = 0.072 min^{-1}<br>AQY = not available	62
CuS/Au<br>[nanoplate/nanoparticle]	photocatalytic degradation of	300-1400 nm	rate constant = 0.37 min^{-1}<br>AQY = not available	63

[none]	methylene blue			
$W_{18}O_{49}$-Au<br><br>[nanobundle-nanoparticle]<br><br>[none]	photocatalytic<br><br>nitrophenol<br><br>hydrogenation	white light	rate constant = not available<br><br>AQY = not available	64

[a]yield was calculated by the authors by using relevant data provided in the paper.

[b]AQY of rhodamine B degradation was defined as the percentage of the rate of concentration change of rhodamine B to the power of irradiation.

4. Supplementary references

1. Gotoh, T. Copper sulfide dendrites prepared by sulfur gas heat treatment of copper. *Phys. Status solidi A* **214**, 1700621 (2017).

2. Živković, A., Roldan, A. & de Leeuw, N. H. Tuning the electronic band gap of Cu_2O via transition metal doping for improved photovoltaic applications. *Phys. Rev. Mater.* **3**, 115202 (2019).

3. Saadeldin, M., Soliman, H. S., Ali, H. A. M. & Sawaby, K. Optical and electrical characterizations of nanoparticle Cu_2S thin films. *Chin. Phys. B* **23**, 046803 (2014).

4. Li, J.-F., Zhang, Y.-J., Ding, S.-Y., Panneerselvam, R. & Tian, Z.-Q. Core–shell nanoparticle-enhanced raman spectroscopy. *Chem. Rev.* **117**, 5002-5069 (2017).

5. Creighton, J.A. & Eadon, D.G. Ultraviolet–visible absorption spectra of the colloidal metallic elements. *J. Chem. Soc., Faraday Trans.* **87**, 3881-3891 (1991).

6. Mulvaney, P., Liz-Marzán, L.M., Giersig, M. & Ung, T. Silica encapsulation of quantum dots and metal clusters. *J. Mater. Chem.* **10**, 1259-1270 (2000).

7. Oldfield, G., Ung, T. & Mulvaney, P. Au@SnO_2 core–shell nanocapacitors. *Adv. Mater.* **12**, 1519-1522 (2000).

8. Underwood, S. & Mulvaney, P. Effect of the solution refractive index on the color of gold colloids. *Langmuir* **10**, 3427-3430 (1994).

9. Seh, Z. W. *et al.* Janus Au-TiO_2 photocatalysts with strong localization of plasmonic near-fields for efficient visible-light hydrogen generation. *Adv. Mater.* **24**, 2310-2314 (2012).

10. Sun, H. *et al.* Controllable growth of Au@TiO_2 yolk–shell nanoparticles and their geometry parameter effects on photocatalytic activity. *New J. Chem.* **41**, 7244-7252 (2017).

11. Wan, X. *et al.* From core-shell to yolk-shell: Keeping the intimately contacted interface for plasmonic metal@semiconductor nanorods toward enhanced near-infrared photoelectrochemical performance. *Nano Res.* **13**, 1162-1170 (2020).

12. Zhang, L., Blom, D. A. & Wang, H. Au–Cu_2O core–shell nanoparticles: A hybrid metal-semiconductor heteronanostructure with geometrically tunable otical poperties. *Chem. Mater.*

23, 4587-4598 (2011).

13. Lou, Y. *et al.* Evaluation of the photoinduced electron relaxation dynamics of $Cu_{1.8}S$ quantum dots. *Phys. Chem. Chem. Phys.* **5**, 1091-1095 (2003).

14. Shaaban, E., Li, G. Probing active sites for carbon oxides hydrogenation on Cu/TiO_2 using infrared spectroscopy. *Commun. Chem.* **5**, 32 (2022).

15. Docao, S., Koirala, A.R., Kim, M.G., Hwang, I.C., Song, M.K., Yoon, K.B. Solar photochemical–thermal water splitting at 140 °C with Cu-loaded TiO_2. *Energy Environ. Sci.* **10**, 628-640 (2017).

16. Rao, C.N.R., Dey, S. Solar thermochemical splitting of water to generate hydrogen. *PNAS* **114**, 13385-13393 (2017).

17. Huang, H., Shi, R., Zhang, X., Zhao, J. Su, C., Zhang, T. Photothermal-assisted triphase photocatalysis over a multifunctional bilayer paper. *Angew. Chem.* **133**, 23145-23151 (2021).

18. Yu, X., Bi, J., Yang, G., Tao, H., Yang, S. Synergistic effect induced high photothermal performance of Au nanorod@Cu_7S_4 yolk–shell Nanooctahedron particles. *J. Phys. Chem. C* **120**, 24533-24541 (2016).

19. Sun, M., Fu, X., Chen, K., Wang, H. Dual-plasmonic gold@copper sulfide core–shell nanoparticles: Phase-selective synthesis and multimodal photothermal and photocatalytic behaviors. *ACS Appl. Mater. Interfaces* **12**, 46146-46161 (2020).

20. Li, Y. *et al.* Coupling resonances of surface plasmon in gold nanorod/copper chalcogenide core−shell nanostructures and their enhanced photothermal effect. *ChemPhysChem* **19**, 1852-1858 (2018).

21. Tao, F. *et al.* From CdS to Cu_7S_4 nanorods via a cation exchange route and their applications: Environmental pollution removal, photothermal conversion and light-induced water evaporation. *ChemistrySelect* **2**, 3039-3048 (2017).

22. Cao, Y. *et al.* Rattle-type Au@$Cu_{2-x}S$ hollow mesoporous nanocrystals with enhanced photothermal efficiency for intracellular oncogenic microRNA detection and chemo-photothermal therapy. *Biomater.* **158**, 23-33 (2018).

23. Ma, S. *et al.* Controlled growth of CdS–$Cu_{2-x}S$ lateral heteroshells on Au nanoparticles with improved photocatalytic activity and photothermal efficiency. *J. Mater. Chem. A* **7**, 3408-3414 (2019).

24. Shanmugam, V. *et al.* Oligonucleotides—assembled Au nanorod-assisted cancer photothermal ablation and combination chemotherapy with targeted dual-drug delivery of doxorubicin and cisplatin prodrug. *ACS Appl. Mater. Interfaces* **6**, 4382-4393 (2014).

25. Hans, E.A.D.R., Regulacio, M. D. Dual plasmonic Au−$Cu_{2-x}S$ nanocomposites: Design strategies and photothermal properties. *Chem. Eur. J.* **27**, 11030-11040 (2021).

26. Ivanchenko, M., Jing, H. Smart design of noble metal–copper chalcogenide dual plasmonic heteronanoarchitectures for emerging applications: Progress and prospects. *Chem. Mater.* **35**, 4598-4620 (2023).

27. Ma, L. *et al.* Pt decorated (Au Nanosphere)/(CuSe ultrathin nanoplate) tangential hybrids for efficient photocatalytic hydrogen generation via dual-plasmon-induced strong Vis–NIR light absorption and interfacial electric field coupling. *Sol. RRL* **4**, 1900376 (2020).

28. Li, D., Yu, S.-H. & Jiang, H.-L. From UV to near-infrared light-responsive metal–organic framework composites: Plasmon and upconversion enhanced photocatalysis. *Adv. Mater.* **30**, 1707377 (2018).

29. Shi, A., Li, H., Yin, S., Zhang, J. & Wang, Y. H_2 evolution over g-C_3N_4/Cs_xWO_3 under NIR light. *Appl. Catal. B* **228**, 75-86 (2018).

30. Gao, J. *et al.* Integrated p-n/schottky-junctions for a high near-infrared photocatalytic H_2 production upon CdZnS/CoP/CoO ternary hybrids with steering charge transfer. *Fuel* **333**, 126331 (2023).

31. Zhang, Y. *et al.* Single near-infrared-laser driven Z-scheme photocatalytic H_2 evolution on upconversion material@Ag_3PO_4@black phosphorus. *J. Chem. Eng.* **375**, 121967 (2019).

32. Zhang, X., Xiao, J., Hou, M., Xiang, Y. & Chen, H. Robust visible/near-infrared light driven hydrogen generation over Z-scheme conjugated polymer/CdS hybrid. *Appl. Catal. B* **224**, 871-876 (2018).

33. Cheng, W. *et al.* Synergetic enhancement of plasmonic hot-electron injection in Au cluster-nanoparticle/C_3N_4 for photocatalytic hydrogen evolution. *J. Mater. Chem. A* **5**, 19649-19655 (2017).

34. Xu, Y., Du, C., Zhou, C. & Yang, S. Ternary noble-metal-free heterostructured NiS–CuS–C_3N_4 with near-infrared response for enhanced photocatalytic hydrogen evolution. *Int J. Hydrog. Energy* **45**, 4084-4094 (2020).

35. Tang, J.-Y., Guo, R.-T., Pan, W.-G. & Zhou, W.-G. Fabricated noble-metal free Co_2B/g-C_3N_4

photocatalyst with 2D/2D structure achieved remarkable water splitting performance from visible to near-infrared wavelengths. *Fuel* **333**, 126280 (2023).

36. Elbanna, O., Zhu, M., Fujitsuka, M. & Majima, T. Black phosphorus sensitized TiO_2 mesocrystal photocatalyst for hydrogen evolution with visible and near-infrared light irradiation. *ACS Catal.* **9**, 3618-3626 (2019).

37. Ma, M.*, et al.* Broadened photocatalytic capability to near-infrared for CdS hybrids and positioning hydrogen evolution sites. *Appl. Catal. B* **325**, 122327 (2023).

38. Gong, S. *et al.* Noble-metal and cocatalyst free W_2N/C/TiO photocatalysts for efficient photocatalytic overall water splitting in visible and near-infrared light regions. *J. Chem. Eng.* **405**, 126913 (2021).

39. Zhu, M., Zhai, C., Fujitsuka, M. & Majima, T. Noble metal-free near-infrared-driven photocatalyst for hydrogen production based on 2D hybrid of black phosphorus/WS_2. *Appl. Catal. B* **221**, 645-651 (2018).

40. Lian, Z. *et al.* Plasmonic p–n junction for infrared light to chemical energy Conversion. *J. Am. Chem. Soc.* **141**, 2446-2450 (2019).

41. Hong, I., Chen, Y.-A., Hsu, Y.-J. & Yong, K. Triple-channel charge transfer over $W_{18}O_{49}$/Au/g-C_3N_4 Z-scheme photocatalysts for achieving broad-spectrum solar hydrogen production. *ACS Appl. Mater. Interfaces* **13**, 52670-52680 (2021).

42. Hou, Y. *et al.* Layered nanojunctions for hydrogen-evolution catalysis. *Angew. Chem. Int. Ed.* **52**, 3621-3625 (2013).

43. Yang, S. *et al.* Size effect of CoS_2 cocatalyst on photocatalytic hydrogen evolution performance of g-C_3N_4. *J. Colloid Interface Sci.* **635**, 305-315 (2023).

44. Li, N., Fan, H., Zhao, W., Gao, Y. & Ge, L. 2D/0D plasmonic CuSe/CdS for efficient photocatalytic hydrogen activity via strong Vis-NIR light and interfacial effect. *Appl. Surf. Sci.* **590**, 153028 (2022).

45. Chu, J., Han, X., Yu, Z., Du, Y., Song, B. & Xu, P. Highly efficient visible-light-driven photocatalytic hydrogen production on CdS/Cu_7S_4/g-C_3N_4 ternary heterostructures. *ACS Appl. Mater. Interfaces* **10**, 20404-20411 (2018).

46. Sun, Z., Liu, X., Yue, Q., Jia, H. & Du, P. Cadmium sulfide nanorods decorated with copper sulfide via one-step cation exchange approach for enhanced photocatalytic hydrogen evolution under visible

light. *ChemCatChem* **8**, 157-162 (2016).

47. Wang, W.*, et al.* Edge-enriched ultrathin MoS_2 embedded yolk-shell TiO_2 with boosted charge transfer for superior photocatalytic H_2 evolution. *Adv. Funct. Mater.* **29**, 1901958 (2019).

48. Wang, X., Li, Y., Li, T. & Jin, Z. Synergistic effect of bimetallic sulfide enhances the performance of CdS photocatalytic hydrogen evolution. *Adv. Sustain. Syst.* **7**, 2200139 (2023).

49. Chen, Y., Qin, Z., Wang, X., Guo, X. & Guo, L. Noble-metal-free Cu_2S-modified photocatalysts for enhanced photocatalytic hydrogen production by forming nanoscale p–n junction structure. *RSC Adv.* **5**, 18159-18166 (2015).

50. Liu, S. *et al.* CdS-$Cu_{1.81}S$ heteronanorods with continuous sublattice for photocatalytic hydrogen production. *Appl. Catal. B* **303**, 120909 (2022).

51. Vamvasakis, I., Trapali, A., Miao, J., Liu, B., Armatas, G. S. Enhanced visible-light photocatalytic hydrogen production activity of three-dimensional mesoporous p-CuS/n-CdS nanocrystal assemblies. *Inorg. Chem. Front.* **4**, 433-441 (2017).

52. Liu, G., Zhao, L., Ma, L., Guo, L. Photocatalytic H_2 evolution under visible light irradiation on a novel $Cd_xCu_yZn_{1-x-y}S$ catalyst. *Catal. Commun.* **9**, 126-130 (2008).

53. Ran, J.*, et al.* $NiPS_3$ ultrathin nanosheets as versatile platform advancing highly active photocatalytic H_2 production. *Nat. Commun.* **13**, 4600 (2022).

54. Yendrapati, T. P., Gautam, A., Bojja, S.& Pal, U. Formation of ZnO@CuS nanorods for efficient photocatalytic hydrogen generation. *Sol Energy* **196**, 540-548 (2020).

55. Guo, S., Li, X., Li, J., Wei, B. Boosting photocatalytic hydrogen production from water by photothermally induced biphase systems. *Nat. Commun.* **12**, 1343 (2021).

56. Gao, D., Xu, J., Wang, L., Zhu, B., Yu, H., Yu, J. Optimizing atomic hydrogen desorption of sulfur-rich NiS_{1+x} cocatalyst for boosting photocatalytic H_2 evolution. *Adv. Mater.* **34**, 2108475 (2022).

57. Shi, X.*, et al.* Protruding Pt single-sites on hexagonal $ZnIn_2S_4$ to accelerate photocatalytic hydrogen evolution. *Nat. Commun.* **13**, 1287 (2022).

58. He H*, et al.* MoS_2/TiO_2 Edge-on heterostructure for efficient photocatalytic hydrogen evolution. *Adv. Energy Mater.* **6**, 1600464 (2016).

59. Sen, S., Shyamal, S., Mehetor, S.K., Sahu, P., Pradhan, N. Au-$Cu_{2-x}Te$ Plasmonic heteronanostructure photoelectrocatalysts. *J. Phys. Chem. Lett.* **12**, 11585-11590 (2021).

60. Ivanchenko, M., Nooshnab, V., Myers, A.F., Large, N., Evangelista, A.J., Jing, H. Enhanced dual plasmonic photocatalysis through plasmonic coupling in eccentric noble metal-nonstoichiometric copper chalcogenide hetero-nanostructures. *Nano Res.* **15**, 1579-1586 (2022).

61. Chen, K. *et al.* Tunable charge transfer and dual plasmon resonances of Au@WO_{3-x} hybrids and applications in photocatalytic hydrogen generation. *Plasmonics* **15**, 21-29 (2020).

62. Ma, L. *et al.* Growth behavior of Au/$Cu_{2-x}S$ hybrids and their plasmon-enhanced dual-functional catalytic activity. *CrystEngComm* **21**, 5610-5617 (2019).

63. Basu, M., Nazir, R., Fageria, P., Pande, S. Construction of CuS/Au heterostructure through a simple photoreduction route for enhanced electrochemical hydrogen evolution and photocatalysis. *Sci. Rep.* **6**, 34738 (2016).

64. Xu, Y. *et al.* Dual-plasmon-enhanced nitrophenol hydrogenation over $W_{18}O_{49}$–Au heterostructures studied at the single-particle level. *Catal. Sci. Technol.* **13**, 1301-1310 (2023).
